# Unpacking the packaged optical fiber bio-sensors: understanding the obstacle for biomedical application

**DOI:** 10.3389/fbioe.2024.1401613

**Published:** 2024-07-31

**Authors:** Aidana Bissen, Nigara Yunussova, Zhuldyz Myrkhiyeva, Aiganym Salken, Daniele Tosi, Aliya Bekmurzayeva

**Affiliations:** ^1^ National Laboratory Astana, Nazarbayev University, Astana, Kazakhstan; ^2^ School of Engineering and Digital Sciences, Nazarbayev University, Astana, Kazakhstan; ^3^ School of Sciences and Humanities, Nazarbayev University, Astana, Kazakhstan; ^4^ Spectrum International School, Astana, Kazakhstan

**Keywords:** optical fiber, biosensor, packaged, microfluidic, wearable

## Abstract

A biosensor is a promising alternative tool for the detection of clinically relevant analytes. Optical fiber as a transducer element in biosensors offers low cost, biocompatibility, and lack of electromagnetic interference. Moreover, due to the miniature size of optical fibers, they have the potential to be used in microfluidic chips and *in vivo* applications. The number of optical fiber biosensors are extensively growing: they have been developed to detect different analytes ranging from small molecules to whole cells. Yet the widespread applications of optical fiber biosensor have been hindered; one of the reasons is the lack of suitable packaging for their real-life application. In order to translate optical fiber biosensors into clinical practice, a proper embedding of biosensors into medical devices or portable chips is often required. A proper packaging approach is frequently as challenging as the sensor architecture itself. Therefore, this review aims to give an unpack different aspects of the integration of optical fiber biosensors into packaging platforms to bring them closer to actual clinical use. Particularly, the paper discusses how optical fiber sensors are integrated into flow cells, organized into microfluidic chips, inserted into catheters, or otherwise encased in medical devices to meet requirements of the prospective applications.

## 1 Introduction

An optical fiber-based biosensor (OFB) is a biosensor which employs an optical fiber as a transducing platform and a biological recognition element as a ligand (Monk and Walt, 2004). Packaged biosensors are biosensors which are put into microfluidic devices, organized into lab-on-a-chip (LOC), integrated into catheters, or otherwise encased in a platform for various applications including *in situ* use. A proper packaging approach is frequently as challenging as, if not more difficult than, the sensor architecture itself. The packaging must shield the sensor and related cables from the environment while still allowing appropriate access to the environment to perform the measurements (French et al., 2005). As a result, solutions for optical component encapsulation and packaging should be explored to match the demands of the final device, namely, by keeping sensing characteristics (Leitão et al., 2022). There is an excellent review paper on the recent progress on the use of optical fiber sensor (OFS) for *in vivo* applications (Bartnik et al., 2024). Given the importance of the topic, some review papers have dedicated a separate section to the packaging of OFS (Leitão et al., 2022). Other papers focus on one type of platform, namely, microfluidic devices or wearable devices or limit their works on one type of OFS such as photonic crystal cavities (Ricciardi et al., 2015; Zhang et al., 2018) or plasmonic sensors (Kim, 2012), the type of analytes (Liao et al., 2019; Yuan et al., 2023), integration of optical fibers with metallic structures and dielectric/semiconductive nanostructures to produce lab-on-fiber technology (Ricciardi et al., 2015). However, there is no work that encompasses all available physical platforms for packaging of all types of OFS for biomedical purposes. Therefore, this work focuses mainly on external platforms for packaging OFS such as flow cells, microfluidic chips, catheters, needles, and wearable devices. The review is divided into six main sections starting with the overview of OFS which were packaged for biomedical applications. The review then highlights different packages focusing on their fabrication, design, and materials. There is also a section dedicated to the analytical performance of such bio-sensors. Then the authors will discuss some important characteristics which are crucial for packaged OFB to meet clinical demand.

## 2 Optical fiber sensors

The working principle of OFB is based on the transmission of light through an optical fiber (silica glass or plastic) to the analysis site (Mehrvar et al., 2000). A glass optical fiber consists of a narrow cylinder called a core which is doped with elements such as Ge to increase its refractive index (RI) (having guided field), a cladding that surrounds the core (having exponentially decaying evanescent field) and a buffer coating (protective coating). Since the core has an RI n_1_ which is higher than the RI of the core (n_2_), the light that entered the core stays inside the material because of total internal reflection and is therefore transmitted forward (Leung et al., 2007; Correia et al., 2018).

**FIGURE 1:**
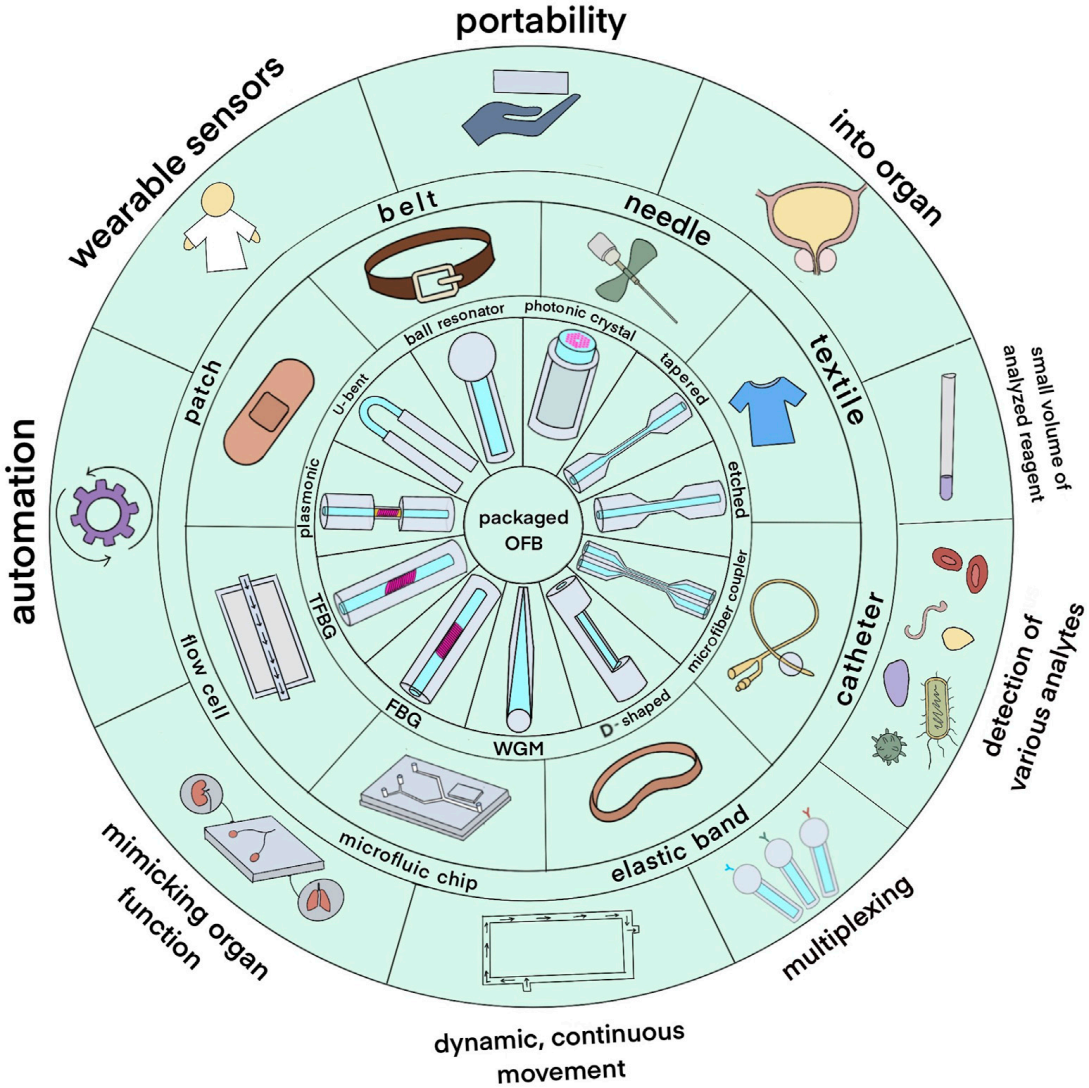
An overview of packaged optical fiber bio-sensors. The inner circle depicts the types of optical fiber sensors; the middle circle shows the types of packages used to integrate the sensors while the outer circle shows potential application areas.

Using optical fiber as a transducer offers such advantages as having low detection limit, low cost, chemical inertness, wide range of surface modification techniques that can be applied and the potential to be used for remote sensing (Chryssis et al., 2005; Leung et al., 2007). Moreover, they have a possibility of being miniaturized, used in *in vivo* applications, and being multiplexed to detect several analytes simultaneously and are immune to electric or magnetic interference (Mehrvar et al., 2000; Marazuela and Moreno-Bondi, 2002). A variety of OFS were integrated into different packaging systems including fiber Bragg grating (FBG) sensors, U-bent, D-shaped, tapered sensors, optical microfiber coupler (OMC), ball resonator, whispering gallery mode (WGM), long-period fiber grating (LPFG) sensors as displayed in the inner circle of Figure 1. Sensing region can be either on the tip of the fiber or along its length. Table 1 shows how these sensors are usually fabricated and some peculiarities of the packaging due to the type of sensors.

**TABLE 1:** Optical fiber sensors integrated with external packaging: sensor and packaging peculiarities.

Fiber optic sensor type	Sensor fabrication	Sensor/packaging shape peculiarities	Ref
PLASTIC OPTICAL FIBER (POF)			
U-bent	- The fiber is declad and this region is bent to form U-shaped geometry - Decreased incident angle of guided light in the bent region which results in higher penetration depth; - Glass fibers are brittle to bend; mostly plastic fibers are used	- POF can resist smaller bend radii than glass OF - POF diameter: 900–980 µm - For 980 µm fiber: polished for a length of 10 mm and for a depth of roughly 150 µm - Waveguide width 200 mm - U-bend portion curvature diameter 1 mm - Microchip with 200 mm and 500 mm channel widths - Flow channel for 980 µm fiber 9.5 mm × 2 mm × 1 mm	Yanase et al. (2010), Aray et al. (2016), Chauhan et al. (2016), Khatri et al. (2018)
Unclad POF	- Made of multimode plastic-clad silica optical fiber - The cladding (2 cm) is removed by CO_2_ laser engraving - Metal (usually gold 40–50 nm) coating	- Diameter 830 µm (core 400 μm, cladding 430 µm) or 600 µm - Unclad area 2 cm - A microfluidic channel for *730* µm coating diameter fiber: (L×W×D) 4 cm × 900 μm × 900 µm or 800 μm × 800 µm	Tseng et al. (2007), Hsu et al. (2011), Verma and Gupta (2013)
D-shaped (side-polished)	- Cladding and part of the core (2–10 µm away from the core) is removed by cutting, grinding - Using etching or ablation - A D-shaped structure with a flat sensing surface when looking at the cross section is formed - Usually fabricated from fibers with a softer material - including MOF and PCFs - But glass fiber can also be used	- Polish length can be 50 mm - Polish depth about 7 mm - For a 125/62.5 µm multimode fiber: channel width of 180 μm, and height of 250 µm	Wang et al. (2021c), Bo et al. (2023)
Evanescent wave all-fiber (tapered/etched plastic)	- Made of plastic-clad step-index silica optical fiber - Cladding is stripped away to expose the core	−5 cm core-exposed region at the tip of the fiber	Wang et al. (2015)
PLASTIC and GLASS OPTICAL FIBERS			
SMS	- Produced by splicing SMF-MMF-SMF - Need to carefully align the fiber for correct mode coupling to MMF and to avoid misalignment - MMF cladding region is removed by chemical etching (HF)	- Length of multimode fiber was 30 mm	Kaushik et al. (2019)
STMS	- Consists of SMF-TNCF-MMF - TNCF 125 μm tapered	- Diameter: 27 μm - Length: 244 μm	Wang et al. (2023)
GLASS OPTICAL FIBERS			
Tapered	- Produced by heating and pulling the fibers - Usually core and cladding diameters are reduced proportionally - Etching, polishing and focused ion beam etching middle portion of the fiber (the cladding is thinned) can also be used	- The tapered regions can have diameter of tens of microns - Tapered region of 15 mm + 5 mm transition region from both sides	Savin et al. (2000)
Ball resonator	- Produced by aligning two SMF, splicing, and heating two SMF - Fabricated using a CO_2_ laser splicer with high laser power	- A spherical tip with a diameter of 450–600 μm at the end of SMF - Stem made of SMF 125 µm - Fits into 20 G catheter	Bekmurzayeva et al. (2022)
FBG	- Fabricated by inscribing periodical refractive index modulation in the core of the SMF - Differ in their central wavelength, and peak reflectivity	- Usually 125 µm in diameter, core is 8.2 µm - Cylindrical shape - The length of the grating can be 10 mm	Vavrinsky et al. (2022)
TFBG/Plasmonic TFBG	- Similar to FBG but the grating is tilted - The tilt angle dictates which cladding modes are excited and therefore the operating range of the sensor - Usually is gold coated - The sensing region can be 2 cm long	- Made of standard SMF - Microchannel (W×D): 200 µm × 150 µm - Hollow cylindrical needle: internal/external diameter of 1.2 mm and 1.6 mm	Guo et al. (2016)
Optical microfiber coupler	- Double twisting two optical fibers in parallel and fixing on the elongation stage - Hydrogen flame to create waist	−0.6–1.2 µm waist diameter - Two SMF surrounding the waist (500 µm)	Zhou et al. (2018)
WGM HGMS	- Fusions splicing of fused silica capillary with SMF - Tapering region: fusion collapse zone between hollow capillary and solid optical fiber for reduction of the angle of collapse zone and improvement of the coupling efficiency	- HGMS 63–75 µm diameter - Fused silica capillary: internal/external diameter of 30/120 μm	Niu et al. (2022)

FBG–fiber Bragg grating; HGMS - Hollow glass microspheres; MMF–multi-mode fiber; MOF - micro-structured optical fibers, SMF–single-mode fiber; SMS - single mode-tapered multimode-single mode; STMS - SMF-TNCF-MMF-SMF; TNCF - thin no-core fiber; TFBG–tilted FBG; WGM–whispering gallery mode.

### 2.1 Fiber Bragg grating (FBG) sensors

FBG is an intrinsic fiber element inside a short segment of an optical fiber that has a certain range of reflected and transmitted wavelengths of light. This is achieved by periodic modulation of the RI inside the core by illuminating it with an ultraviolet (UV) light to build grating structure. As light enters the core of the fiber and travels through FBG structure, the wavelength is selectively reflected by Bragg gratings (Mishra et al., 2011). Packaged FBG sensors used gratings as active sensing elements in several works. FBG at a much lower wavelength was generated by Arasu et al. using white light source with further gold coating for achieving surface plasmon resonance (SPR) effect (Arasu et al., 2016). The gratings generated by this way refract the light to the cladding, but the light does not escape from the fiber due to total internal reflection (TIR) producing evanescent waves which then can be used as SPR. Gold coated tilted FBG (TFBG) packaged in a hollow cylindrical needle were used as a sensing tool for cancer biomarker testing in tissue (Ribaut et al., 2017). Another study used FBG as a second element: temperature compensation during DNA measurements with an active element being micro-structured optical fiber (MOF).

### 2.2 D-shaped/side polished sensors

The D-shaped plastic optical fiber (POF) sensors have also been developed and packaged for biosensing (Hu et al., 2023). The sensors were produced using multimode POF which are first fixed either by encapsulating the fiber within a solid block of resin or by placing it in the V-grove with the shape that exactly matches a semi-circle and then polishing it, so it exposes the core, resulting in a D-shaped profile. The polishing can be done using a computer numerical control (CNC) micro-milling machine or by manually grinding using a sandpaper (Bo et al., 2023; Seitz, 2024). The D-shaped POF for respiration monitoring was integrated the sensor into an elastic band. Another POF with a side-polish length of 50 mm and a polish depth of 7 mm was used to detect anti-coagulant in a sample circulation unit (Wang Y. L. et al., 2021).

D-shaped sensors were further modified in some cases for producing biosensors. For instance, the exposed core is drilled with microholes that have a diameter of around 600 µm and a depth of 500 µm. The sensor contains a SPR probe and a capture platform including three microholes containing receptor solutions. Molecular interactions may be detected by observing the changes in light propagation in the POF core caused by the binding of target molecules in the microholes (Seitz, 2024)*.* D-shaped fiber was also used as platform for subscribing gratings with subsequent etching to produce highly sensitive RI sensors (Tahhan et al., 2017).

### 2.3 Tapered optical fiber sensors

One of the simplest ways for fabricating optical fiber sensor components is based on tapering a relatively small piece of optical fiber with length varying from sub-millimeter to tens of millimeters (Korposh et al., 2019). Tapered optical fiber provide several advantages for sensor development, including wide evanescent fields, flexibility, and compactness and is produced by heating a specific segment of the fiber while drawing the two ends of the fiber together (Kamil et al., 2019; Korposh et al., 2019). For some very fragile sensors such as tapered optical fibers, microfluidic chip or a flow cell is needed for protection of the sensor (Tian et al., 2011). The geometrical structure of tapered optical fiber is nonuniform within the sensing region, liquid handling needs an improvement because during each measurement it is crucial to ensure that the liquid is removed completely from the optical fiber. With multiple measurement, it is difficult to handle the tapered optical fiber due to its delicate structure (Abdul Hamid et al., 2017).

The taper fiber or microwire employed single-mode fibre-28 (SMF-28) with two 12 mm concatenated tapers separated by 10 mm. The first down taper zone had no full cladding-core mode recoupling and provided an evanescent field at the slop. Both propagating core mode and stimulated cladding modes displayed a differential phase shift in the up-taper region, demonstrating interference at the transition. If transition slopes were fast enough, cladding-core mode recoupling was poor, and light passed through. A concatenated tapered microwire’s output spectrum demonstrated spectral modulation with regularly spaced notch times inversely linked to tapered region separations (Shrivastav et al., 2019)*.*

The thin-core microfiber (TCF) was developed by splicing it between two SMFs using a fusion splicer. A nonadiabatic taper was formed on the TCF by using a fusion device to induce an arc discharge. The taper was improved by subjecting it to heat from a hydrogen flame and stretching the fiber using an optical fiber pulling machine. The ultimate TCF possessed a waist diameter of around 6.4 µm and a waist length of about 2,357 μm, leading to a transmission spectrum with a free spectral range of 35.28 nm (Chen et al., 2023).

### 2.4 Other types of optical fiber sensors

A ball resonator sensor (Shaimerdenova et al., 2020) is another type of optical fiber packaged for biosensing. It operates as a weak interferometer, and exhibited superficial fringe patterns and a spectrum that showed characteristics to a random signal. It can be manufactured using a commercially available CO_2_ splicing machine. The sensitivity of the resonators’ RI might be assessed by measuring either the shift in wavelength or the change in amplitude. The fiber possesses a ball lens on its tip with diameter of around 500 nm.

Another sensor is a single-multi-mode fiber optic coupler (SMFC) which was employed to transmit incident light and capture Fresnel reflected light, simplifying the system by eliminating the requirement for optical separation components and improving light transmission efficiency. This layout significantly enhanced the sensitivity of the all-fiber Fresnel reflection microfluidic biosensor (FRMB). In the bio-probe made of multimode fiber, the incident light that enters the fiber is reflected at the far end, and the strength of the reflected light changes depending on the local RI. The binding of target biomolecules to the bio-probe leads to a rise in RI, resulting in a decrease in the intensity of reflected light. This allows for the identification of target molecules without the need for labeling, based on their concentration (Xu et al., 2021). A Fabry-Pérot (F–P) sensor was developed by employing two-photon photopolymerization (TPP) technique to construct a microcantilever structure by photolithography located at the tip of an optical fiber (Li et al., 2022).

## 3 Packaging optical fiber sensors and biosensors

### 3.1 Type of packages

Optical fiber sensors in different packages for various applications are shown in the middle layer of Figure 1 and vary from flow cells and microfluidic chips to medical and wearable devices. Flow cells are typically utilized where a constant sample flow is applied to the sensor surface for applications requiring continuous monitoring and dynamic measurement (Liu et al., 2015; Arasu et al., 2016). The enormous use of microfluidic chips in biosensing applications prevails because they can accommodate large volumes of fluids with high precision (Bo et al., 2023). In this same microfluidic chip, optical fiber sensors can be integrated to monitor real-time changes in the properties of the fluid being handled (Niu et al., 2022). Furthermore, optical fiber sensing is a known technology incorporated within syringes, catheters, and other medical diagnostic and treatment monitoring devices (Sypabekova et al., 2022; Zhou et al., 2022). These devices can be pre-made or custom-modified to include optical sensing capabilities (Bekmurzayeva et al., 2022). Wearable optical fiber sensors can be sewn into fabrics or flexible bands to form a smart patch or elastic band. It is designed to monitor physiological parameters such as heart rate, respiration, and body glucose level by continuous monitoring (Wang Y. L. et al., 2021; Lo Presti et al., 2022; Han et al., 2023).

### 3.2 Fabrication of packages: different technologies

The development of OFS packaging relies on specific design requirements and intended applications. For microfluidic chips, photolithography and soft lithography methods are used (Soni et al., 2021). A silicon wafer is first used to create detailed microchannel patterns, which serve as molds to cast polydimethylsiloxane (PDMS). This layer is then bonded to a glass slide, forming the final device. Flow cells are fabricated through precision machining or micro-molding, often using transparent materials like glass and polymethyl methacrylate (PMMA) (Liu et al., 2015; Arasu et al., 2016). In most medical devices, optical fibers are passed through pre-existing channels or grooves in needles and catheters (Fan et al., 2016). Structures can be modified to fit application needs; for instance, needles can be thermo-molded and encased in thermoplastic, with a window for sensor exposure (Ribaut et al., 2017). Modifications include attaching microdialysis membranes for continuous protein measurement (Ngernsutivorakul et al., 2017) or making holes in catheters for blood-mimicking fluid experiments (Myrkhiyeva et al., 2024). In wearables, optical fiber sensors are integrated into textiles or flexible polymers using sewing, knitting, and lamination techniques (Han et al., 2023).

#### 3.2.1 Photolithography and soft lithography

In microfluidic chip fabrication, the leading technology is usually spin photolithography (Niu et al., 2022). For the master mold, negative photoresist SU-8 is used. The SU-8 solution undergoes spin-coating on a silicon wafer at 1,000 rpm, followed by soft-baking at 65°C for 10 min and 95°C for 30 min. Photolithography converts the microfluidic chip pattern into imaging data, which is then loaded onto a DMD-based maskless lithography system. The SU-8 photoresist is patterned with a UV light source at 365 nm, producing master molds of the microfluidic channels (Yin et al., 2016). PDMS molding is done using a SYLGARD 184 kit with a curing agent, and the PDMS layer is bonded onto a glass slide after oxygen plasma treatment (Yin et al., 2016; Niu et al., 2022).

Soft lithography uses non-photolithographic molding for microfabrication (Liu et al., 2015). A smart microfluidic chip with optical micro/nanofibers have microchannels that were fabricated using soft lithography (Liu et al., 2018; Zhang et al., 2020). In brief, a thin layer of PDMS was bonded on top of the soft lithography pattern to seal the microchannel (Soni et al., 2021). PDMS microchannels are fabricated by molding on a glass slide (Bo et al., 2023). This method creates an undisturbed sensing environment, encapsulating the sensor and preventing environmental interference (Chen et al., 2023).

#### 3.2.2 CNC/laser machining

Another approach of microchip fabrication involves mechanical methods such as machining. The programmed equipment graves the channels and chambers at different power rates by CNC or Laser (Shakarim et al., 2022). For instance, a PCR-SPR microdevice was designed in PMMA substrates through CNC milling (Nguyen et al., 2017). Using PMMA for microdevice fabrication is straightforward and effective for biosensing (Hussein et al., 2023). Patterns in the PMMA substrate are created using a commercial CO_2_ laser engraving machine, with laser power and travel speed determined by CAD software (Shakarim et al., 2022). Access holes for fluid entry and exit are also drilled by a CO_2_ laser. PMMA layers are bonded using chloroform-based adhesive under low temperatures and pressures.

Manufacturing of a microfluidic chip can be done by hot embossing: pre milling and ultraprecision diamond tooling to create two molds—one with the inverted microchannel design and the other with inverted fiber grooves (Ottevaere et al., 2016). The two-sided hot embossing is performed to obtain a microstructured PMMA layer having an 80 µm wide microchannel and grooves for fiber alignment. Spin-coated UV curing adhesive bonds layers, resulting in a robust, low-cost microfluidic chip for dual-fiber optical trapping.

#### 3.2.3 3D printing

One of the fast methods to fabricate various chips is 3D printing. Scientists (Monaghan et al., 2016; Polley et al., 2019) elaborate on the use of the stereolithography (SL) for the preparation of microfluidic chips. The process involves the production of LOC devices with built-in sensing optics for real-time analysis. It is performed with a 3D systems Viper Si2 stereolithography system comprising a UV laser, which can deliver power of 100 mW. The primary material – transparent Accura resin, - can withstand various organic solvents. The SL system allows producing several design iterations at one run, thereby permitting a comprehensive estimation and optimization of the chip design.

Significant steps involved in 3D printing of the microfluidic chips include chip design, preparation of the SL system, printing of the chip, embedding of optical fibers, sealing of channels, and characterization and testing (Bayram et al., 2018; Wang H. et al., 2021). The chip’s design with the help of CAD software includes various geometries of channels and their sizes to be used for analysis in different analytical needs.

#### 3.2.4 Wearable devices

The fabrication of textile-based wearable sensors, particularly those used for sweat collection and analysis, involves various steps (Wang Y. L. et al., 2021). Textile substrates such as polyester or cotton are cleaned by soaking in ethanol and deionized water, followed by drying. The surface is then modified with perfluoro decanethiol (PFDT) to achieve hydrophobicity. Patterned hydrophilic areas are created using oxygen plasma cleaning through a paper mask. The wearable sensor is assembled by punching holes in a PDMS layer to create reservoirs, securing the patterned textile and PDMS layer onto the skin, and integrating optical fiber sensors into the PDMS reservoirs (Liu et al., 2022; Zhao et al., 2023).

For smart patches based on FBG technology, the fabrication involves designing and 3D printing a mold, mixing, and degassing silicone rubber, embedding an FBG sensor in the mold, curing the silicone rubber, and encapsulating the sensor between fabric liners (Gong et al., 2019).

### 3.3 Design of packages and used materials

Different fiber optic sensor packages vary in design and materials based on their functionalities and purposes. Flow cells, for instance, can be rectangular, cylindrical, cuvette-shaped, or spiral to enhance path length or fiber positioning. They may include static or inline mixers, observation windows for spectroscopic analysis, electrodes for electrochemical studies, and membranes for specific reactions (Liu et al., 2015; Kaushik et al., 2019; Shen et al., 2020; Soni et al., 2021). A spiral flow cell for cell culture could incorporate membrane filtration to prevent clogging (Yin et al., 2021).

Microfluidic chips, typically thin and flat with etched microchannels, can feature complex shapes with multiple inlets, outlets, and chambers. They facilitate droplet generation, efficient fluid mixing through micromixers, and fluid control via micromechanical valves. (Liu et al., 2018; Wang H. et al., 2021). Medical device packages, such as long flexible tubes, vary in diameter and tip design like catheters. They might include expandable balloons for angioplasty, side ports for drug delivery, pressure or temperature sensors, and multiple lumens for delivering various medications. For example, a urinary catheter might have a simple drainage port, while a central venous catheter could have multiple lumens (Poeggel et al., 2015; Zhou et al., 2022). Syringes range from pre-filled types for convenience to insulin syringes for precise dosing and catheter-tipped syringes for accurate placement (Wu et al., 2015). Needles, used in various medical applications, come in different bevel angles, lengths, and gauges, including hypodermic, spinal, butterfly, and blunt needles (Hu et al., 2018; Hsieh et al., 2022). Wearable fiber optic probes, such as smart patches and elastic bands, are designed to be flexible and conform to body contours, integrating sensors for vital signs, biochemical markers, and drug delivery via microneedles. Some also include electrodes for muscle or nerve stimulation and wireless communication for data transmission (Lo Presti et al., 2022).

Packaging materials for optical fiber sensors need to be transparent, biocompatible, and mechanically stable. Table 2 shows common materials used for building microfluidic chips for packaged OFB. PMMA is preferred for its optical clarity and mechanical properties, while PDMS is favored for microfluidic chips due to its flexibility and ease of fabrication (Abdul Hamid et al., 2017; Shakarim et al., 2022). Transparency is crucial for biochips used in cell viability studies, often using materials like glass or water-transparent membranes (Huang et al., 2020). For fluorescence-based biosensors, materials with low background fluorescence are chosen (Ngernsutivorakul et al., 2017).

**TABLE 2:** Some materials used for making microfluidic chips integrating optical fiber bio-sensors.

Material	Fabrication method	Advantages	Disadvantages	Ref
PDMS	SLA	- High precision and resolution - Complex structures - Biocompatible - Flexible	-Expensive and time-consuming fabrication process - Post-processing process - Not heat resistant	Amin et al. (2016), Olmos et al. (2019), Raj M and Chakraborty (2020), Kulkarni et al. (2022)
Photo-polymer resin	3D Printing	- Rapid prototyping - Customization through software - Ease of fabrication - High resolution of material - Widespread	- May have limited biocompatibility	Amin et al. (2016), Olmos et al. (2019), Xu et al. (2022)
PMMA	CNC Machining, Laser cutting	- Transparency for optical applications - Chemical resistance	- Brittle material, limited to certain applications - Cost - Not heat resistant - Not biocompatible	Hong et al. (2010), Amin et al. (2016), Kulkarni et al. (2022)

PDMS, polydimethylsiloxane; PMMA, Polymethyl Methacrylate; SLA, Stereolithography.

When it comes to medical devices, the material is usually biocompatible and approved for use in humans. These materials include stainless steel for needles. Silicone and rubber are used in industries to make syringes and catheters for medical purposes (Zhou et al., 2022). However, the main challenge with these devices is that their structure should sometimes be modified to both fit the optical fiber sensor and the final application. Another thing to consider when it comes to needles/catheters is their gauge/diameter. Thus, a larger fiber (400 µm) where encased in 21-gauge needles while fibers with smaller diameter (100 µm) fibers in 31-gauge needles (Hsu et al., 2017). This also applies to the intended blood vessel (for *in vivo* applications): smaller blood vessels will require larger gauge needles/catheters and *vice versa*.

### 3.4 Packaging optic fiber bio-sensors

#### 3.4.1 Integrating with flow cells

**FIGURE 2:**
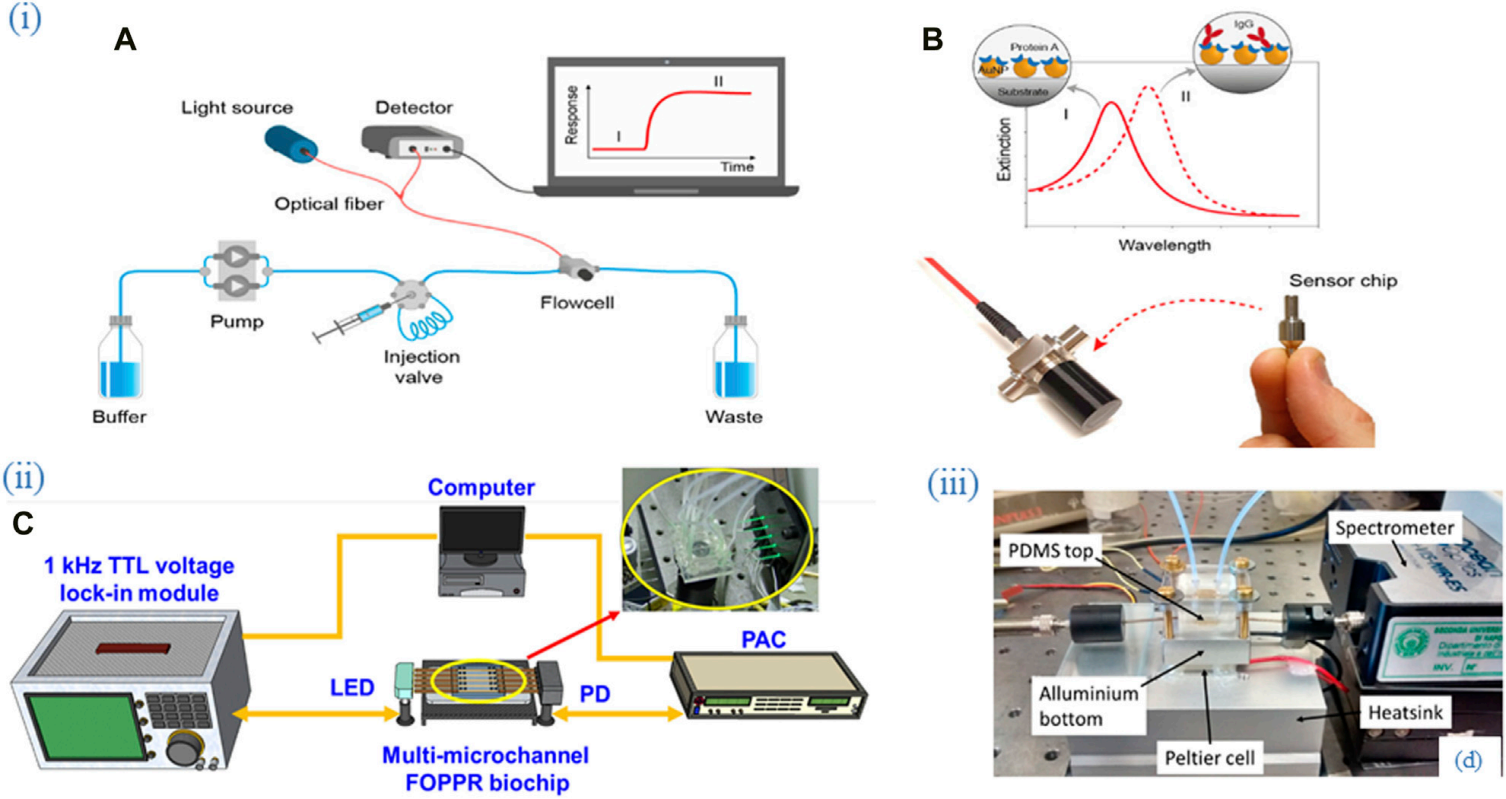
Flow cells as part of experimental setups employing optical fiber biosensors. (i) **(A)** An experimental setup of the packaged sensor system. **(B)** Nanoplasmonic sensor chips were functionalized with Protein A for specific recognition of IgG. Adapted from (Tran et al., 2020) (ii) **(C)** An experimental setup consisting of the multi-microchannel sensing system. The multi-microchannel biochip was composed of two PMMA plates, a cover and a bottom plate, with dimensions of 40 mm × 30 mm × 4 mm and fabricated by using a CO_2_ laser. Adapted from (Chiang et al., 2020), (iii) **(D)** An experimental setup consisting of the thermo-stabilized flow cell system. The aluminum bottom part of the flow cell is mounted in thermal contact with a Peltier cell (20 mm) and a thermistor is inserted into the lateral hole. Adapted from (Cennamo et al., 2016). All images licensed under Creative Commons CC BY 4.0.

Functionalization fibers with ligands and/or incubating biosensors with analytes is usually done in laboratory dishes of different size including microtubes (Matjasec and Donlagic, 2017; Ribaut et al., 2017; Lee et al., 2018), microtube caps (Bekmurzayeva et al., 2021; Bekmurzayeva et al., 2022), Petri dish (Luo et al., 2018), glass/capillary tube (Tedeschi et al., 2003; Tahhan et al., 2017; Sypabekova et al., 2022). In some cases, special chambers/tanks were built for holding the sensor and incubating with the analyte of interest. Some examples of flow cells used for experimental setups of optical fiber biosensors is shown in Figure 2. During the measurements using a flow cell, the upstream leading-in fiber of the taper tip is mounted on a holder, and the fiber tip is freely placed on a lift platform with a resolution of 10 nm to facilitate the bending process and measurement under an optical microscope (Bao et al., 2017). A commercially available fiber-optic XY positioner was used to build a vertical optical stage to hold and move a U-bent fiber for the measurement of sucrose in juices (Chauhan et al., 2016).

A simple glass flow cell was built to hold SPR-based OFB covered with MoS_2_ nanosheets to detect bacterial cells (Kaushik et al., 2019). Teflon fiber bath was used to hold polymer optical fiber during the detection of analytes (Cao et al., 2018). A fiber optic plasmonic sensor was integrated into a custom 3D-printed flow cell for continuous detection. This setup allowed different solutions to flow over the sensor surface, enabling real-time biosensing. Multiple pumps ensured controlled chemical flow, preventing contamination and bubbles, demonstrating the sensor’s high sensitivity and specificity for biosensing applications (Polley et al., 2019). The fluid flow was controlled by a peristaltic pump.

In some cases, special platforms were built not only for analyte measurement but also for sensor fabrication and functionalization. A multimode tapered fiber was functionalized directly in an acrylic platform with a groove; analyte (anti-virus antibody) detection was also performed in the same platform (Wang et al., 2015). A dynamic etching setup was used to control the etching of the fiber – remove its cladding and expose its core for sensing purposes. A special vial has been designed and fabricated to preserve the precise alignment of the axis of rotation of optical fiber and the vial during the rotation and extraction of the fiber. Etching in a dynamic setup it was possible to obtain fibers that had reproducible nanotips. The researchers were able to get the probes having different angle and shape of the cone and surface roughness (Barucci et al., 2015).

A flow cell holding an LPFG sensor was used to measure antibodies released by cancer cells, indicating hypoxia. One end of the sensing probe was connected to a light source and the other to an interrogator (Tyagi et al., 2018). A flow cell with an unclad optical fiber coated with molecularly imprinted polymers (MIP) selectively detected glucose, with water washing between measurements for high specificity (Azad et al., 2022).

A fiber-optic cholesterol biosensor using localized and propagating surface plasmons on an unclad fiber in a flow cell detected cholesterol. Three probes with different ad-layers (silver coating, graphene oxide nanosheets, silver nanoparticles) and cholesterol oxidase modifications were compared (Semwal and Gupta, 2018). A PDMS flow cell with ports for solution flow was used for real-time fibrinogen detection, fitted with a metal-coated fiber and an SPR sensor head using a multimode optical fiber (Nguyen et al., 2015). A U-grove flow cell held a U-bent LPFG sensor for measuring triacylglycerides, placed in an aluminum block with a thermocouple for analyte concentration measurement (Baliyan et al., 2016). A chitosan-coated U-bent LSPR sensor on a glass support, with an LED and portable spectrometer, moved by an X-Y positioner into a proteinase-free reservoir, measured amyloid proteins (Khatri et al., 2018).

Another example of an OFB integration with the flow cell can be the work of Usha et al. where a sensor was created to detect p-cresol, a common environmental toxin using MIP as a ligand (Usha and Gupta, 2018). Flow of the sample (artificial urine containing p-cresol) through the cell allows a constant interaction between the sensor and the target molecules enabling continuous monitoring and real-time detection. These flow systems served for preserving the delicate structure of some biosensors and allowed dynamic/real-time sensing; they can be considered as prototypes for more complex platforms like microfluidic chips.

#### 3.4.2 Integrating with microfluidic chips

Microfluidics has been defined as fluid manipulation structures and channels which are micrometer in size; compared to macroscale structures the liquid behaves differently in such small channels (Drug Delivery Devices and Therapeutic Systems, 2021). Microfluidics were historically constructed for electrophoresis to achieve superior resolution over chromatography and provide faster microscale diagnostics. Key contributions include Tiselius’ 1930s moving boundary method and advancements by Haugaard and Kroner in the 1940s and 1950s, with recent interest driven by the low cost and ease of mPADs for point-of-care diagnostics (Nanthasurasak et al., 2017). Microfluidic systems are like microscopic biology and chemical labs (Kulkarni et al., 2022). Microfluidic technology has enabled the fabrication of miniature systems, and portability may be utilized for POC testing applications (Liu et al., 2020). The integration of microfluidics with biosensors creates a potent tool that can replace bulky conventional equipment by combining chemical and biological components onto a single platform (Luka et al., 2015). Sample manipulation, effective and speedy response, mobility, operational transparency, controllability, precision, and stability are all significant aspects of microfluidic devices used for biological analysis (Derkus, 2016; Kulkarni et al., 2022).

Conventional optical devices used in biomicrofluidics rely largely on bulky optical components such as lenses, waveguides, and lasers in the form of off-chip microscopy. There is still a demand for low-cost, sensitive, and portable optically micro engineered spectroscopic detection devices (Liao et al., 2019). The microelectronics sector offers efficient methods for fabricating microdevices on silicon wafers. While keeping the same sensitivity as similar systems, on-chip or partially on-chip optics has advantages in terms of footprint, cost, and timeline (Bates and Lu, 2016).

**FIGURE 3:**
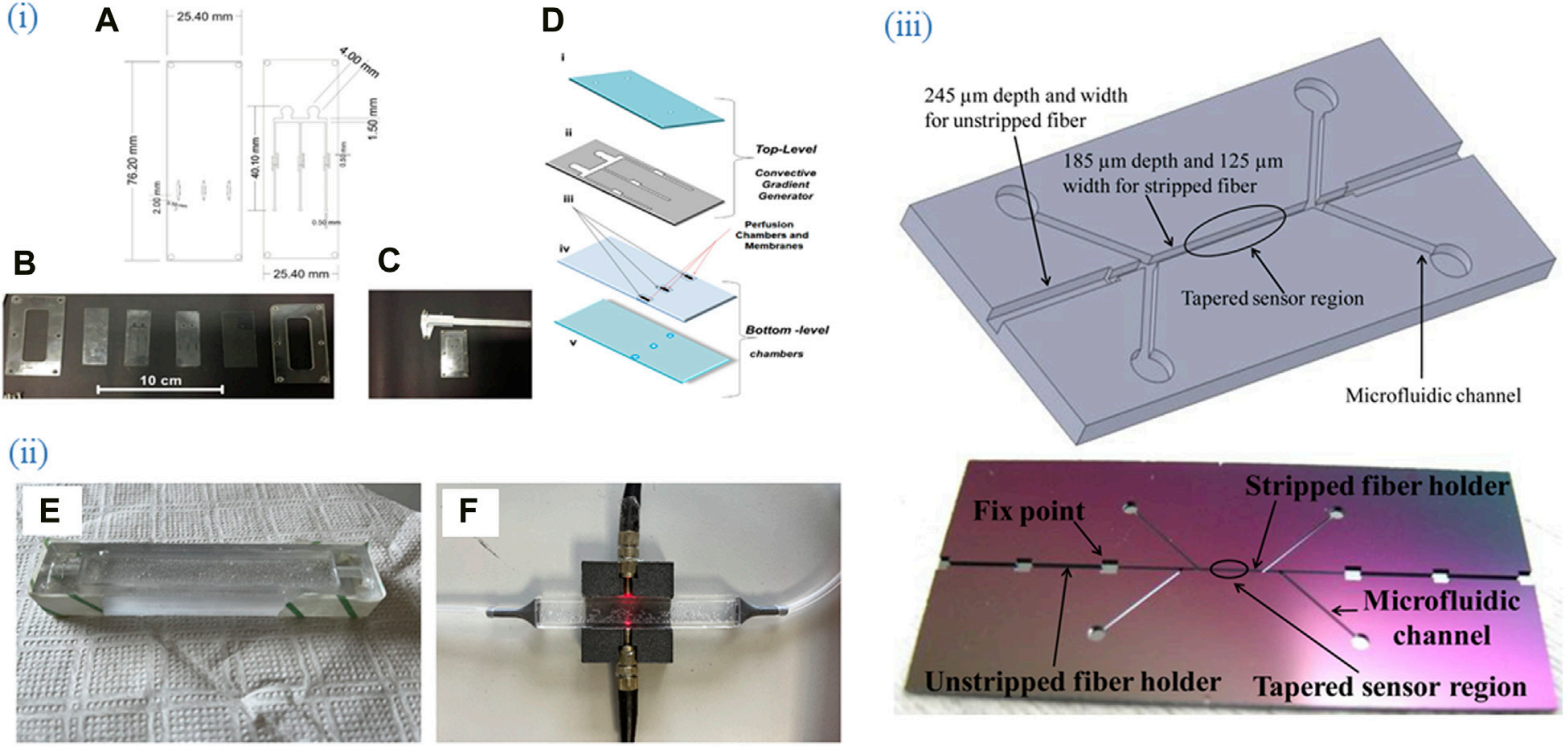
Various designs and fabrication approaches of microfluidic chips. (i) **(A)** Project of the two PDMS layers of the microfermentor. **(B)** Layers and acrylic molds before the fermentor assembly. **(C)** Micro Fermenter before the insertion and sealing of the optical fibers. **(D)** schematic diagram of the micro fermenter: top-level (i) cover (glass), and (ii) microchannels (PDMS laminated); and bottom-level (iii) membranes (polycarbonate), (iv) intermediate sheet with holes (PDMS laminated), and (v) base (glass). Adapted from (Soares et al., 2019). (ii) Representation of the molding process and completion of the microchip with mounting OFS. **(E)** PDMS in mold before heat processing and top layer bubble removal. **(F)** PDMS sensor after fabrication with light and pneumatic circuit. Adapted from (Sannino et al., 2023). (iii) (upper) Chip structure and (lower) Real etched chip. The four microfluidic channels branches were designed for inlets and outlets of bio-target and fluid, respectively. Adapted from (Tian et al., 2011), All images licensed under Creative Commons CC BY 4.0.

Figure 3 shows various designs and fabrication approaches of microfluidic chips. Early integration of OFS into chips was driven by the need for high-performance and miniaturized fiber optic sensing systems. Application-Specific Photonics Integrated Circuits (ASPICs) enabled the development of compact, robust, and highly sensitive FBG interrogator systems. These integrated photonic chips, such as those used in the Space Gator project, provided significant advancements in terms of size, weight, power consumption, and reliability, making them suitable for demanding applications in space and other harsh environments (Evenblij and Leijtens, 2017). The system was further automated (Bayram et al., 2018) by including easy integration and assembling. The paper details a microfluidic flow cytometer built using glass micropipettes and a 3D-printed aligner. It achieves hydrodynamic focusing with varied sheath/sample flow pressures, tested with uniform-sized microparticles. The setup, integrating fiber optics, enhances performance and stability, is ultra-low cost (under $1), and is easy to assemble (under 10 min). This design excludes bulk optics, simplifying integration. These works, however, are based on fluorescently labeled reagents and are thus not label-free. The portable system allowed a fast detection of multiple analytes using OFB.

Wang et al. used a reusable aptamer-based evanescent wave all-fiber (EWA) biosensor for the detection of a common food-contaminating mycotoxin – ochratoxin A) in a flow cell (Wang et al., 2015). The separated toxin was pumped through the flow cell with the integrated EWA sensor inside. The actual sensing of the analyte by the ligand occurred in a static condition. The flow system was used for pumping the analyte through the sensor and for studying the performance of the biosensor after regeneration.

*In situ* and *in vivo* applications can also be made possible for fiber optic interferometer sensors. The use of a biosensing probe based on fiber optic interferometer (FOI) provides continuous protein monitoring with high sensitivity, immediate signal response, and repeatable detection capabilities (Hu et al., 2023). The perfusion-based micro opto-fluidic system (PMOFS) setup includes a FOI sensor, a UV fiber, and a microfluidic system. The resulting PMOFS apparatus is a needle-type probe, which offers the capability for real-time protein detection, surface regeneration, and repeatable use in *in situ* measurements.

In the study of Shen et al. integrating the TFBG with the partially gold coated TFBG microfluidic platform benefits in compact size that fits into microfluidic channels (Shen et al., 2020). Additionally, depicted high sensitivity to both flow rate and flow direction is promising while remains relatively temperature insensitive by referencing the core mode resonance. The only problem with this integration process is that it coats the layer with gold: difficult to get a uniform and accurate coat of gold, which may compromise the consistency and reliability of sensor performance. In this regard, the integration process is supposed to be very complex and may result in inconsistencies in the coating quality.

Tapered optical fiber sensors become very fragile once fabricated; for their protection as well as for biotesting purposes, the sensor was integrated with a microfluidic chip that had different width of the microchannels depending on the region of the optical fiber that fitted inside them (Li et al., 2018). Geometry of the microchannels (profile and depth) were precisely controlled by microelectromechanical systems (MEMS) fabrication, namely, photolithography and deep dry etching resulting in an accuracy of 2 μm.

A sensor consisting of an unclad region of plastic optical fiber coated with thin gold film was placed inside a thermo-stabilized microfluidic platform that was made of two parts: an aluminum bottom which houses the tip of the sensor, thermistor and an upper PDMS part with a channel and inlet/outlet for fluid flow. A Peltier cell is placed between these two parts for temperature stabilization. The platform was used not only for thermal and mechanical stability during the measurement of the analyte of interest, but also for studying the immobilization of ligands (anti-CRP antibodies) in flow conditions. The biosensor detected CRP with a limit of detection (LOD) of 0.009 mg/L in serum in a label-free manner (Aray et al., 2016).

The integration of OFS with microchips demonstrates significant advancements in biosensing capabilities, exemplified by a variety of innovative approaches. Zhou et al. (Zhou et al., 2018) developed a label-free immunosensor using an optomechanical crystal sensor for detecting cardiac troponin I (cTnI), a biomarker for acute myocardial infarction. This biosensor, featuring a PDMS chamber for sample delivery, exhibited ultrahigh sensitivity, reaching 91777.9 nm/RIU near the turning point, making it highly applicable in clinical diagnostics due to its simplicity, quick response time, and ease of handling. Another approach involved a WGM fiber probe with a hollow glass microsphere (HGMS), packaged in a PDMS microfluidic channel with a double-Y structure, achieving an LOD of 0.59 ng/mL and a detection resolution of 1.2 fg/mL (Niu et al., 2022). Further innovations include an optofluidic sensor integrated into a microfluidic chip, functionalized with PEI and single-stranded pDNA, which utilized a peristaltic pump for liquid injection, ensuring precise real-time monitoring of solution temperature fluctuations (Hu et al., 2023).

Microchips create sensitive environment for fiber optic bio-sensors. Guo et al. developed plasmonic TFBG sensors with nanometric coatings for proteinuria detection using microfluidics, achieving a protein concentration sensitivity of 5.5 dB/(mg/mL) and a detection limit of 1.5 × 10⁻³ mg/mL (Guo et al., 2016). Jia-Huan Qu et al. developed a POC biosensor for therapeutic drug monitoring of adalimumab using fiber optic surface plasmon resonance (FO-SPR) with self-powered microfluidics, achieving an LOD of 0.35 μg/mL, requiring only 1 μL of plasma (Qu et al., 2022). Esposito et al. introduced an LPFG biosensor for detecting C-reactive protein in serum, enhanced with graphene oxide, and achieving an LOD of 0.15 ng/mL (Esposito et al., 2021). The device’s working point is tuned to the mode transition region by chemical etching, improving sensitivity while maintaining spectral features. Vogelbacher et al. introduced a silicon nitride (SiN) optical waveguide Mach-Zehnder interferometer (MZI) integrated with an optically pumped organic solid-state laser (OSSL) for cost-effective, compact, and sensitive POC diagnostics, demonstrating high functionality and integration density (Vogelbacher et al., 2022). The integration of OSSL eliminates the need for precise alignment, which is a common challenge in biosensors. Lastly, Noor et al. developed a PMMA microfluidic chip for packaging optical fibers and detecting fluorescence signals, emphasizing its affordability and customization potential despite challenges like surface roughness and bonding inconsistencies (Hussein et al., n. d.).

Findings by Wang et al., Shakarim et al., and Chang et al. focus on integrating fiber optic sensors into microfluidic chips, each with distinct fabrication methods and advantages (Chang et al., 2022; Shakarim et al., 2022; Wang et al., 2024). Shakarim et al. developed a microfluidic chip for label-free cell viability assays using SPR to detect changes in refractive index (RI), indicating live cells (Shakarim et al., 2022). Wang et al. created a dual-color TIR fluorescence (TIRF) detection platform for multiple fluorescent signals, enhancing sensitivity and multiplexing capabilities (Wang et al., 2024). Chang et al. presented a power-free microfluidic chip for on-site nucleic acid detection using fiber optic particle plasmon resonance (FOPPR), offering high specificity and portability (Chang et al., 2022). SPR-based chips are sensitive and suitable for dynamic studies but require complex fabrication and alignment. TIRF platforms excel in multiplexing and high-throughput analysis but are hindered by background fluorescence and need for sophisticated optics. FOPPR-based chips are easy to construct and portable for point-of-care applications but less sensitive compared to SPR and TIRF. Recent advances include robust sensing materials and multivariate detection capabilities in a single chip, enhancing sensitivity and reducing sample volumes. A microfluidic system with buried optical fibers detected viral pathogens in orchids, incorporating micromixers, microvalves, and micropumps for precise fluid manipulation and minimal human intervention (Lin et al., 2015). An optofluidic biosensor for DNA hybridization and methylation used a tunable mode coupler in an in-line PCF Michelson interferometer, demonstrating high specificity and repeatability with a 5 nM LOD for DNA hybridization (Gao et al., 2016). Both systems integrate microfluidics with optical components for precise fluid handling and detection, emphasizing minimal intervention, high sensitivity, and real-time monitoring.

#### 3.4.3 Integrating with medical or medical-like devices

**FIGURE 4:**
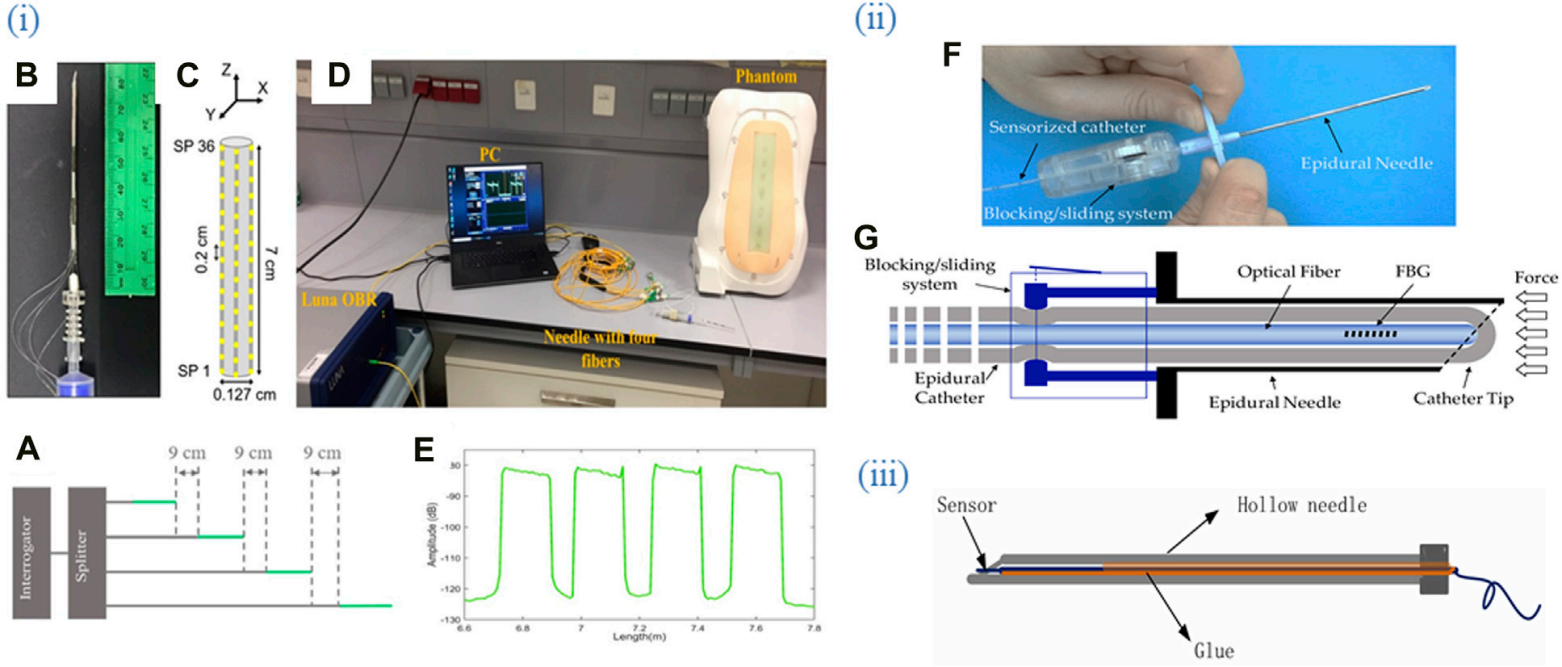
Optical fiber biosensors are packaged into medical devices such as catheters, syringes, and needles. (i) **(A)** Schematic of the multiplexing methodology of the NPDFs. **(B)** The upper view of the fiber mount on the needle. **(C)** The schematic of the fiber arrangement on the needle. **(D)** An experimental setup comprising the fibers glued along the epidural needle and connected to the Luna OBR interrogator via the splitter. **(E)** Backscattering trace of the OBR in proximity of the 4-fiber sensing setup. Adapted from (Amantayeva et al., 2021). (ii) **(F)** The developed guiding device comprises a standard 20 G nylon EC integrated inside the lumen of an epidural needle. **(G)** The FBG is inserted inside the catheter lumen and locked in position. Adapted from (Carotenuto et al., 2018). (iii) Sensor package structure. Integration of OFS into the hollow needle. Adapted from (Zhao et al., 2021). All images licensed under Creative Commons CC BY 4.0.

The next popular packaging approach for optical fiber bio/sensors is using needles, catheters, or similar medical devices as shown in Figure 4. Urodynamic analysis replicates the operation of the urinary system using cycles of filling and emptying the bladder, as a critical diagnostic tool for bladder-related diseases (Poeggel et al., 2015). For biological applications, OFS can replace MEMS transducers which are usually attached to catheters filled with fluid or air and used as pressure sensors in urology. New developments in pressure sensors based on the extrinsic Fabry-Perot interferometer (EFPI) concept have made it possible to produce more compact sensors with the maximum precision. Due to the fiber optic EFPI sensors’ small size, numerous probes can be inserted into a single catheter to detect pressure at various points inside the bladder and urethra without obstructing the passageway.

One more catheter based OFB was developed for real-time observation of chemotherapy-induced tumor apoptosis, the advanced biosensor is designed to be inserted directly into the tumor site via a catheter, allowing for continuous monitoring of cellular responses to chemotherapy (Fan et al., 2016). The catheter-like sensing probe was made of two optical fibers and two microcapillaries connected to an off-the-shelf portable system. The OFB aims to provide *in vivo* monitoring of non-homogeneously distributed apoptotic biomarkers induced by chemotherapy agents. The sensor employs a 2-fluorophore assay system, with phospholipid-conjugated Marina Blue (FluoMb) as a cell distribution indicator and FM 1–43 as an apoptotic activity indicator. The apoptosis detection was based on the ratio of the two fluorophore emissions.

To replace endoscopic methods, a study by Hsin-Yi Wen et al. introduced a nonintrusive approach for biomedical assays using a double helix DNA-shaped optical fiber sensor packaged to a quartz glass tube (Wen et al., 2022). This sensor detects changes in the refractive index (RI) upon binding with gastrin-17 (G-17), a hormone secreted by stomach G-cells, leading to wavelength shifts that enable real-time measurements with high sensitivity. The design’s potential applications in biomedical assays target gastric cancer-related biomarkers.

Parent et al. developed a new guidance system for intra-arterial procedures using optical-frequency-domain reflectometry (OFDR) for continuous 3D shape reconstruction of a catheter within hepatic arteries (Parent et al., 2017). This system enhances navigation through complex vascular structures for trans-arterial chemoembolization interventions by employing UV-exposed fibers to boost Rayleigh scattering. A similar advancement was made with a magnetic position sensor for scanning fiber endoscopes, incorporating a micro-magnet and a Hall sensor to provide real-time feedback and high-resolution OCT images, thereby improving clinical accuracy and stability (Çalıkoglu et al., 2024).

Stephens et al. detailed the integration of fiber Bragg grating (FBG) sensors into needles for precise navigation and localization during biopsies, emphasizing their importance in high-precision medical procedures (Stephens et al., 2021). Comparatively, OFDR-based systems offer continuous data along the fiber length, suitable for complex navigations but requiring sophisticated UV exposure techniques (Gong et al., 2019; Zhao et al., 2023). Magnetic position sensors enhance long-term stability and are ideal for repeatable clinical applications, although they are limited by the need for magnetic components. FBG sensors, while highly accurate, are costly and less flexible than other methods.

Further integration of these sensors into medical devices can be achieved through innovative approaches that combine these sensing techniques or enhance miniaturization and integration. For instance, a plasmonic optical fiber immunosensor was engineered to detect lung cancer biomarker cytokeratin 17 using a specially constructed needle packaging (Ribaut et al., 2017). The package included of a hollow cylindrical needle with an internal diameter of 1.2 mm and an exterior diameter of 1.6 mm. The platform was purposefully engineered to suit the optical fiber sensor, with precise dimensions to guarantee both safety and functioning. The needle tip was shaped by thermo-molding, while the body was formed using extrusion. This platform ensured both safety and functionality, demonstrating the feasibility of precise biomarker identification in soft tissues using plasmonic optical fiber grating immunosensors.

Another study used a plasmonic optical fiber sensor encased in a needle for sensitive dopamine detection (Hu et al., 2018). This biosensor, incorporating a gold-coated tilted fiber Bragg grating (TFBG) and graphene, used a single-stranded DNA aptamer to selectively bind dopamine, enhancing sensitivity and specificity with a lower detection limit of 10^−13^ M. This setup is well-suited for *in situ* monitoring in hard-to-reach locations. Additionally, a fiber sensor was packaged into a 27-gauge stainless steel blunt-tip needle setup involved electrospinning a polymer solution, with fibers deposited on glass substrates and crosslinked to ensure durability (Stephens et al., 2021). This biosensor demonstrated significant advancements in developing affordable, scalable label-free optical biosensors for rapid and sensitive target analyte detection. Finally, an optical fiber biosensor designed for intrathecal catheter integration achieved attomolar-level detection of the cancer biomarker CD44 (Bekmurzayeva et al., 2022). The fiber is designed for insertion into an intrathecal catheter with micrometer-sized holes to allow fluid exchange. This biosensor allows repeated sampling by replacing the fiber, providing high spatial resolution and a low sample volume for clinical applications and POC cytokine detection devices. Another biosensor aimed to detect IL-6 secreted by BV2 cells was also integrated into catheter. This spatially resolved ELISA detects IL-6 with high spatial resolution (200–450 μm) and a sample volume of 1 μL. The catheter’s diameter and fiber difference of 155 μm minimize friction. The fiber captures cytokines, which are detected after incubation and washing (Liu et al., 2017).

#### 3.4.4 Integrating into wearable devices

**FIGURE 5:**
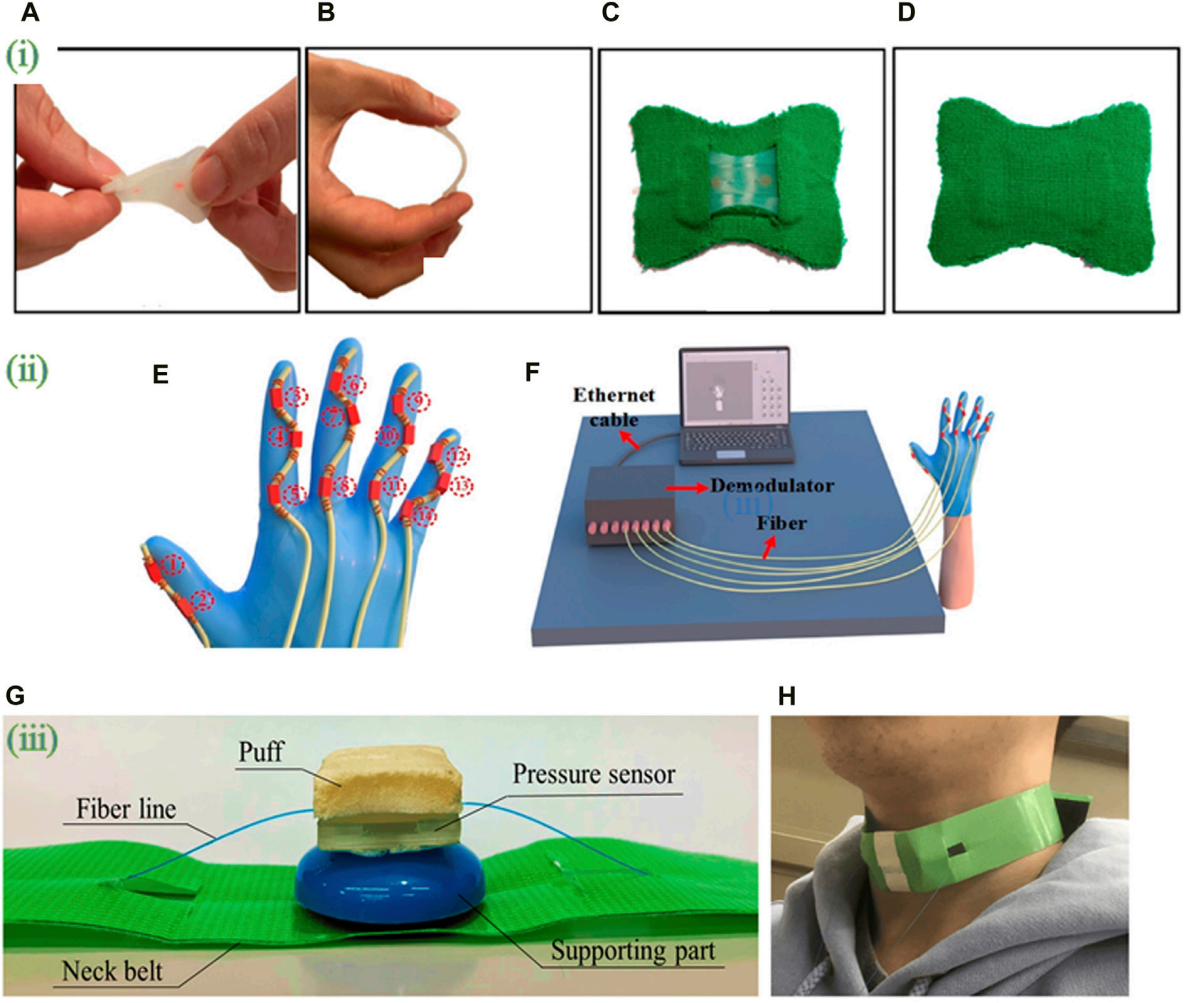
Optical fiber biosensors packaged into wearable devices. (i) **(A)** Twisting and **(B)** bending of the silicone layer embedding the FBG sensor; **(C)** the smart patch backend and **(D)** frontend. Adapted from (Lo Presti et al., 2022). (ii) **(E)** Schematic diagram of FBG sensor integration. A glove is fitted with five serialized sensing arrays containing FBG sensors. **(F)** attitude reconfiguration system comprising five parallel FBG arrays connected to the five channels of the FBG demodulator. Adapted from (Rao et al., 2023). (iii) **(G)** photograph of the wearable swallowing assessment device with fabricated hetero-core fiber-optic pressure sensor attached to a neck belt. **(H)** appearance when a user attaches the wearable swallowing assessment device. The belt was used to apply a stable level of pressure between the larynx and the device. Adapted from (Maeda et al., 2023). All images licensed under Creative Commons CC BY 4.0.

Wearable electronics are becoming more popular as technology advances, devices being miniaturized, improved materials are developed, and the internet is introduced (Bartnik et al., 2024). Fiber optic technology has progressed over time and has gradually been used in textiles in a wearable mode for diverse applications such as communication, display, sensing, and monitoring (Gong et al., 2019; Liu et al., 2022; Zhao et al., 2023). Optical fibers are frequently utilized in wearable sensors, particularly in chest belts, smart clothes, textiles, pillows, and mattresses, among other applications. They are mainly utilized in the form of traditional sensors or interferometric principles like FBG (Vavrinsky et al., 2022). Respiration, heart activity, blood pressure and flow, oxygen saturation, shear stress, mobility, gait, temperature, and electrolyte balance may all be monitored using optical fibers as discreet and flexible systems (Massaroni et al., 2015; Trung et al., 2018). Figure 5 shows examples of OFS packaged into wearable devices.

Wearable sensors are currently available commercially in the form of smart watches, gloves, patches, tattoos, facemasks, wrist bands, clothing, and glucometers (Afsarimanesh et al., 2020; Promphet et al., 2021; Qiao et al., 2022). In contrast to standard optical fibers used for signal transmission, where light is reflected inside the core and emitted at the fiber’s end, optical fibers designed for wearable lighting clothing include tiny holes on the lateral side that penetrate through the cladding to the core (Gong et al., 2019).

As optical fibers are formed of inorganic substances, they have a high biological endurance, however it implies that end-of-life considerations for textiles incorporating optical fiber must be properly examined. Chemical endurance of optical fibers is mostly determined by the materials used in the fiber core, cladding, and coating; biological endurance is exceptional since optical fibers are constructed of inorganic substances (Sousa et al., 2020). Optical fibers mimic textile fibers or yarns, hence the most logical approach to integrate OFS in clothes would be to incorporate the optical fiber into a fabric.

In one study, researchers combined a D-shaped cross-section plastic optical fiber (POF) with an elastic band to create a respiratory sensing system for reliable recording during mobility (Wang Y. L. et al., 2021). The system includes a light source, D-shaped POF sensor on an elastic belt, an optical receiver, and a microprocessor. The elastic belt comprises a D-shaped POF sensor, a plastic belt piece, and elastic cloth.

A grapefruit optical fiber coated with Ag nanoparticles was integrated as a sensitive SERS probe with a wearable Janus fabric for efficient sweat collection. The sharp points of grapefruit optical fibers can pierce clear dressings (Han et al., 2023). Perspiration is drawn into its microchannels by capillary force, with nanoliter amounts filling them entirely. Plasmonic hot regions amplify the Raman signal of sweat components. Sodium lactate and urea can be detected in sweat at levels lower than those in physiological fluids, and the system can analyze actual human perspiration.

Encapsulating the FBG sensor in flexible substrates gives it a skin-like feel and improves user acceptance. Silicon rubber (Dragon SkinTM 20) was used as an encapsulating layer for the FBG sensor to enhance durability and skin adhesion (Lo Presti et al., 2022). A wearable blood pressure monitor utilizing a precise FBG sensor measures the pulse wave signal at the body’s pulsation point, using an optical fiber that reflects light at 1,550 nm (Koyama et al., 2017). The Bragg wavelength shift, indicating blood pressure, can be integrated into wearable items like wristbands or sleeves.

### 3.5 Experimental setups

The setup, especially the optical part of the setup will depend on the sensor type as can be seen from Figures 2–4. Thus, a typical setup for plasmonic TFBG, for example, includes a broadband light source for excitation of the sensor, a linear polarizer and polarization controller for adjusting and orienting the polarization state of the light which passed through the FBG, a sensor and a downstream OSA as an interrogator of tunable linearly polarized light. The inlet and outlet of the analytes in the microfluidic system is before and after the sensor respectively as in these works (Guo et al., 2014; Guo et al., 2016). For LPFG in DCF, a broadband optical source to launch the light and an OSA downstream can be used as in the study by Hsu et al. (Hsu et al., 2011). For a tapered sensor, a setup could include an optical sensing interrogator such as Micron Optics si720 which would excite the fiber from one end and monitor the transmission from the other end of the fiber while the sensing region is integrated into a microfluidic chip (Tian et al., 2011). An optical backscatter reflectometry can be used both to launch the light and measure the reflected light through a ball resonator sensor inserted into a plastic tube via a catheter/cannula system (Zhang et al., 2019). Another option is using a light source-sensor (Verma and Gupta, 2013). An experimental setup for monitoring chemotherapy-induced apoptosis involved a fiber-optic biosensor with a customized sensing probe made of optical fibers and microcapillaries. The system included light sources connected to the optical fibers, a spectrophotometer to analyze the collected spectra, and a syringe pump to deliver the fluorescent dyes (Fan et al., 2016).

Securely fixing sensors is important in the fabrication of packaged biosensors, particularly those integrated into microfluidic chips, to ensure their optimal performance. One of the methods used for this purpose involves using of UV-sensitive adhesives (Guo et al., 2014). Employing a UV-curing adhesive (NOA 61) to secure a WGM fiber probe inside the microfluidic channel used by capillary action, securely attaches the probe and effectively connects the passage to prevent any potential leakage. The WGM fiber probe is firmly placed under UV light to provide a one-stop assay. This method effectively integrates sample handling, reaction, separation, and detection within the adaptable structure of the microfluidic channel (Niu et al., 2022). Similarly, optical fibers are secured in position by using optical glue (NOA 68, Norland) within V-shaped grooves located on 2 bars of the apparatus which have a depth of around 0.3 mm. The glue undergoes polymerization when exposed to UV light, resulting in a solid state within a time frame of about 15–20 min. This process leads to the stable alignment of the fibers (Esposito et al., 2021).

### 3.6 Other assay parameters

**FIGURE 6:**
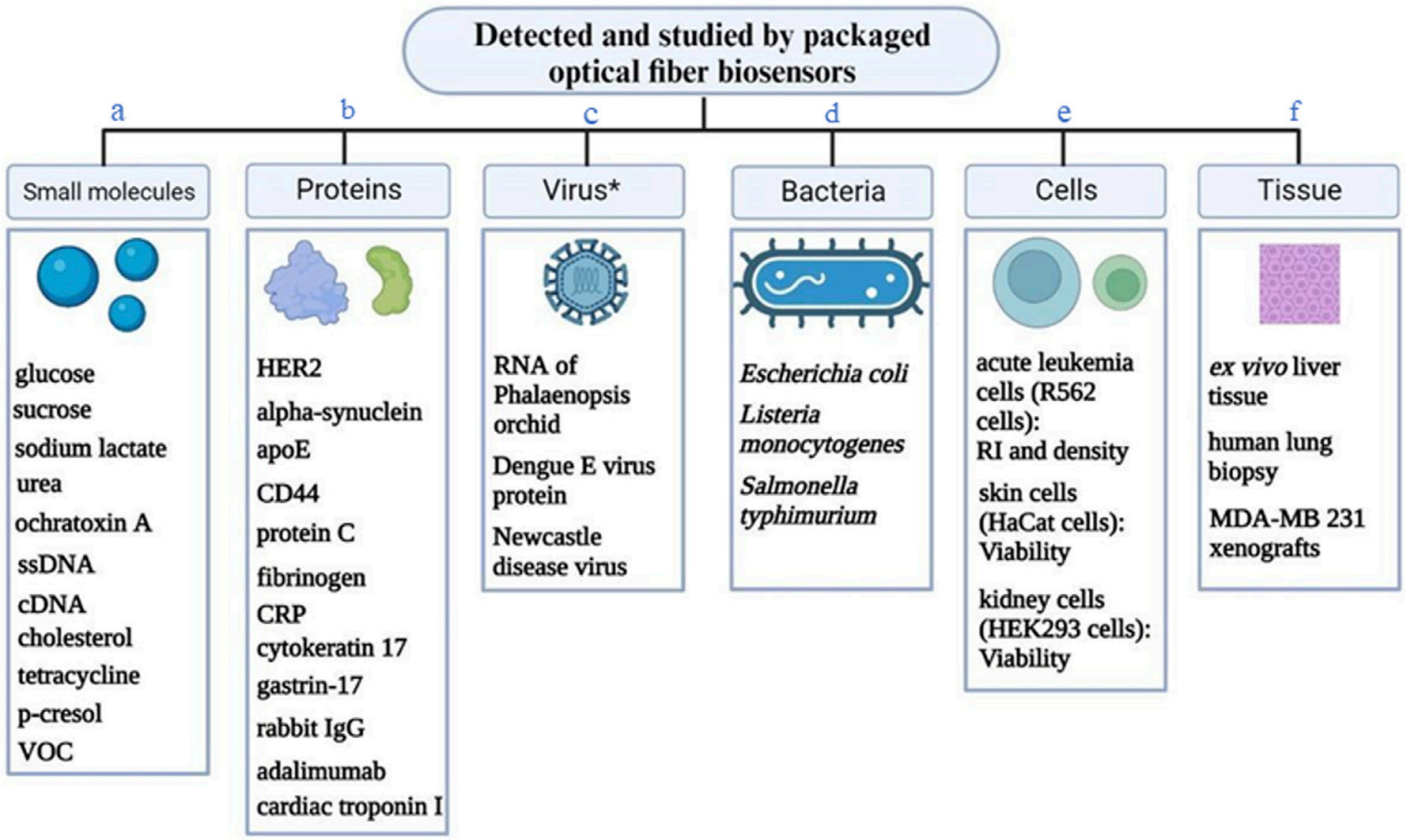
A variety of analytes detected or studied by the packaged optical fiber biosensors. * - virus or viral protein. **(A)** (Tolosa et al., 1999; Tang et al., 2002; Endo et al., 2006; Tseng et al., 2007; Tian et al., 2011; Weidemaier et al., 2011; Evers et al., 2012; Thakkar et al., 2013; Guo et al., 2014; Barucci et al., 2015; Lin et al., 2015; Nguyen et al., 2015; Wang et al., 2015; Amin et al., 2016; Chauhan et al., 2016; Lee et al., 2018; Tyagi et al., 2018; Afsarimanesh et al., 2020; Wen et al., 2022; Kundu et al., 2024); **(B)** (Nguyen et al., 2015; Khatri et al., 2018; Zhou et al., 2018; Tran et al., 2020; Bekmurzayeva et al., 2021; Bekmurzayeva et al., 2022; Qu et al., 2022; Sypabekova et al., 2022; Wen et al., 2022); **(C)** (Hsu et al., 2011; Fan et al., 2016; Mustapha Kamil et al., 2018; Kamil et al., 2019); **(D)** (Verma and Gupta, 2013; Kaushik et al., 2019; Soni et al., 2021); **(E)** (Haddock et al., 2003; Fan et al., 2016; Semwal and Gupta, 2018; Usha and Gupta, 2018; Olmos et al., 2019; Vogelbacher et al., 2022); **(F)** (Evers et al., 2012; Semwal and Gupta, 2018; Qu et al., 2022; Xu et al., 2022).

Representative ligand-analyte pairs which were used in building packaged OFB include antibody-antigen, receptor-ligand (estrogen receptor and estradiol), enzyme-substrate (glucose oxidase-glucose), aptamer-target, ssDNA probe-target DNA, MIP-target. Figure 6 shows the variety of analytes measured by the optical fiber bio-sensors integrated with external packaging. This includes small molecules, proteins, viruses, bacteria, cells and even tissues.

Accurate temperature measurement is crucial in the domain of packed biosensors since it directly impacts the biochemical processes and sensor responses in microfluidic systems. Diverse techniques have been utilized to precisely monitor and regulate temperature in these environments. A technique involves utilizing FBG, which can do detection in real-time and track temperature fluctuations in the solution as it flows through the microchannel. This technology is competent not only in measuring temperature but also in identifying DNA hybridization events (Hu et al., 2023).

Temperature regulation can be achieved by employing a thermistor, thermocouple with an interconnected thermometric feedback control unit which is integrated into the flow cell or microfluidic channel (Aray et al., 2016; Khatri et al., 2018). For example, in a particular design, both bars of the device have a lateral hole with a 2 mm diameter, which is appropriate for accommodating a thermocouple. The thermocouple, located at the bottom bar, is an integral component of a temperature control system. It sends feedback to an electronic driver which in turn regulates the Peltier cells. On contrast, the thermocouple on the upper bar is linked to a thermometric device (Lutron TM-917) that gauges the temperature within the fluidic channel. This configuration is especially vital in experiments that involve solutions sensitive to temperature, as it guarantees that the solutions attain the required temperature specified by the control system, even if they are originally held at lower temperatures, such as in a refrigerator (Esposito et al., 2021).

## 4 Analytical performance of packaged OFB

### 4.1 Sensitivity and limit of detection (LOD)

The change of response for every unit of an analyte concentration for a biosensor is called sensitivity. The sensitivity (s) is calculated as the ratio of the signal amplitude (y) to the unit concentration (x) of the response slope, which is given by the slope of the linear area in the fitting curve. A greater y value for a comparable x value signifies superior sensitivity performance of the biosensor (Prabowo et al., 2021).

Sensitivity of the biosensor highly depends on the type of optical fiber which is employed as a sensor. For instance, a higher sensitivity of Fresnel reflection microfluidic biosensor was due to four grades higher interaction area compared to SMF which in turn leads to increased interaction of the target molecules and the immobilized ligand. Moreover, compact structure of the fiber enhances the effectiveness of optical transmission and collection and lowers light loss (Xu et al., 2021).

For some biosensors, the packaging did not affect its sensitivity but enhanced analyte detection range. The research results by Nguyen *et al.* suggested that the microfluidic chip had a sensitivity that is nearly identical to the LPG glucose biosensor when tested outside the chip (Nguyen et al., 2017). Furthermore, the chip had a broadened detection range spanning from 2 μM to 10 µM. The microfluidic device had the ability to accurately detect glucose concentrations as low as 1 nM. The integration of the sensor into the microfluidic chip increased its detection range from 2 μM to 10 µM (Yin et al., 2016)*.* Upon placement into the microfluidic chip, the response time was significantly reduced from 6 min to 70 s.

One work presented a new sensing chip that incorporated three microholes, which detects variations in light propagation characteristics caused by the binding of targets and receptors, which was likely due to local temperature changes resulting from the binding response. The concentration of receptors in the microholes may be readily increased in contrast to typical sensors where receptors are bound to a solid substrate. This design greatly improves the sensitivity compared to traditional SPR and MIP-based SPR sensors. It enables the detection of interactions between target molecules and receptors at concentrations as low as aM levels, which is equivalent to a few hundred molecules in a 20 µL sample that is applied to the D-shaped plastic POF and diffused into the microcuvettes filled with receptors (Cennamo et al., 2024).

For other packaged OFB, the sensitivity was enhanced by the means of materials. The sensitivity of plasmonic FBG was enhanced using nanostructured graphene oxide coating (Arasu et al., 2016). Titanium dioxide nanostructured metal oxide was coated over the etched D-shaped FBG sensors for the purpose of increasing the sensing area of the sensor which ultimately led to the increase of its sensitivity to RI in a dynamic setup (Tahhan et al., 2017). The experimental findings demonstrated that the SDSMF-LPG biosensor, employing a hybrid sensing film, had the capability to detect glucose concentration with sensitivity, reaching as low as 1 nM (Yin et al., 2016).

According to IUPAC, LOD is defined as the lowest concentration of a substance that can be confidently detected, signified by a specific value that indicates the minimum measurable concentration within a given confidence level (Prabowo et al., 2021). A crucial factor in assessing the performance of DNA biosensors (and other biosensors) was their specific LOD. A lower detection limit is anticipated compared to MIP-based devices due to the potentially higher affinity of bioreceptors in aqueous solutions (homogeneous phase chemical reactions) compared to the binding sites in MIP (adsorption reactions) (Cennamo et al., 2024)*.* Performance-wise, some of such biosensors could achieve very low LOD such was the case in the following studies: 0.24 p.m. (Wang et al., 2023) and 1.87 nM (Hu et al., 2023) for sensing DNA hybridization, detection of proteins at 9.3 fM in dynamic conditions (Bekmurzayeva et al., 2022).

### 4.2 Repeatability and reproducibility

Repeatability/reproducibility of biosensors is crucial for ensuring consistent performance and reliability in various applications. A microfluidic channel, among other advantages, is a useful tool to evaluate the performance of the developed biosensor, namely, testing its repeatability. The movement of the artificial urine sample containing p-cresol through the cell allowed studying the stability and repeatability of the probe. The sensor was also tested in more extreme non-laboratory conditions and was proved to measure p-cresol rapidly and accurately due to the stability of the ligand (Usha and Gupta, 2018). The effect of pH on the performance of a plasmonic biosensor, stability, and repeatability of the biosensor in several cycles of 0 and 10 mM of cholesterol was also performed (Semwal and Gupta, 2018). For assessing the reliability and stability of the sensor in experiments involving RI sensing, identical tests were performed ten times. During these tests, spectral fluctuations were recorded for different RI values. To tackle temperature sensitivity crosstalk to the RI, a matrix was created to assess temperature sensitivity and RI sensitivity simultaneously. The sensor exhibited excellent stability and reliability in repetitive sensing trials, with no impact from liquid velocity and placement angle, thereby showing great potential for on-chip and *in situ* optofluidic detection (Li et al., 2022). The repeatability and reproducibility of the integrated microchannel PCR equipment were comprehensively evaluated resulting in a strong correlation between the fluorescence data and sensor measurements validated the device’s dependable functionality and its capacity for repeated usage with thorough washing (Nguyen et al., 2017).

To evaluate the repeatability of samples, Bo et al., conducted a series of tests where a 10^−7^ mol/L solution of R6G was introduced into a microfluidic channel integrated with a D-shaped optical fiber SERS probe. After recording the R6G Raman signals, the channel was flushed with anhydrous ethanol to clean it, and the process was repeated five times demonstrating reliable repeatability of the microfluidic SERS probe (Bo et al., 2023).

The precision and repeatability of the BP-functionalized sensor was evaluated through a series of stability tests involving tracking the wavelength response of cDNA at a concentration of 1 nM. The results showed that the peak wavelength stabilized approximately 30 min into the measurement. Subsequent readings displayed remarkably consistent wavelength with the maximum discrepancy between these shifts, significantly lower than the typical random wavelength variations of the sensor. This consistency underscores the sensor’s reliable repeatability, affirming its effectiveness for precise applications (Wang et al., 2023).

In a study by Soni et al., the repeatability of microchip fabrications was tested by creating two separate batches of chips, each containing six devices with the same design. These devices were assessed for bulk RI sensitivity by exposing them to various concentrations of glycerol solutions, with the results measured over a consistent flow rate and duration. Results indicated a high degree of uniformity within each chip, with minimal measurement error. However, a notable variation among the devices suggested potential inconsistencies due to the variability in the on-campus fabrication facilities (Soni et al., 2021).

Sensor reproducibility, reusability, and stability were evaluated by testing different sensor chips functionalized and used on various days. Across five independent experiments with at least ten biosensors each, an average absolute resonance shift response was measured indicating a consistent bacteria concentration detection. Up to three regeneration cycles (using 10 mM glycine–HCl pH two solution) was shown to not affect the sensor’s performance. Storing the sensors at 4°C in phosphate-buffered saline (PBS) showed to be the most effective method, maintaining sensor functionality close to that of freshly prepared sensors. In contrast, accelerated aging at 35°C led to an increase in resonance shifts, likely due to antibody precipitation on the plasmonic surface, indicating less stability under these conditions (Soni et al., 2021).

Thus, the following strategies for testing the repeatability of packaged biosensors include: 1) preparing several biosensors and assessing their response to target analytes; 2) regenerating the biosensor’s surface to remove bound analytes, then repeating the measurements over several cycles. This approach ensures that the biosensors maintain consistent performance and reliability across multiple uses, providing a thorough validation of their operational stability and repeatability.

### 4.3 Selectivity and specificity

Specific sensors are those which, in an ideal world, recognize only its target analyte (Peveler et al., 2016). For this, optical fiber sensors, after being fabricated, are functionalized with ligands specific to the target of interest (antibodies, aptamers, molecularly imprinted polymers (MIP) or enzyme). However, achieving this ideal is difficult because of the similarity of analytes or the absence of specific ligands against some targets (Peveler et al., 2016). Being selective is one of the main strong features of biosensors compared to other analytical methods. This property allows the biosensor to discriminate the analyte of interest among other entities in a complex mixture with no pre-separation (Bucur et al., 2021).

The selectivity/specificity of the built biosensors can be measured in several strategies some of which were used in case of packaged OFB as shown in Table 3. One of the strategies is incubating fully functionalized biosensor with other analytes and measuring the obtained signal change. Usually, these negative controls are related to the target analyte in some way or another (also expresses protein on its surface; can be widely found in the biological fluid where target is tested) and the same concertation is measured. The obtained signal change (wavelength shift or amplitude change) is expected to be lower when controls are measured. This might be due to external interference or a minimal amount of non-specific adhesion (Song et al., 2016). Highly selective interferometry biosensor to detect a common pesticide was developed thanks to MIP. The probe demonstrated maximum selectivity for parathion methyl (PM) samples due to the complementary PM sites in the MIP nanoparticle layer, which restricts binding of other molecules (Shrivastav et al., 2019)*.* Some works used two types of strategies (Luo et al., 2018; Bekmurzayeva et al., 2022) to study specificity of the built biosensors.

**TABLE 3:** Strategies for studying specificity/selectivity of packaged optical fiber biosensors.

Optical fiber sensor/Ligand	Target analyte	Negative control/sensor	Why used	Ref.
Strategy 1: Measuring signal change to negative controls				
LPFG CA9 mono-clonal antibodies	A549 cells (express carbonic anhydrase-IX (CA9))	Other carcinoma cell lines: JIMT-1, HepG2, PANC-1, and SW620 (human breast, hepatocellular, epitheloid and colon carcinoma respectively) MRC-5 human fibroblast	Other carcinoma cell lines which also have the expression of the target molecule under target condition (hypoxic) and a cell line which has no expression the protein in both conditions (hypoxic and normoxic)	Tyagi et al. (2018)
Interferometer + FBG pDNA	scDNA	non-scDNA sc-DNA-a sc-DNA-b	scDNA with another sequence of nucleotides scDNA with one and four base mismatch compared to target scDNA	Hu et al. (2023)
TFBG Cytokeratin 17 (CK17) antibodies	CK17	FBS	Mixture of different proteins that can bind the surface	Ribaut et al. (2017)
Ball resonator CD44 antibodies	CD44 protein	Prostate specific antigen	Another cancer biomarker Also present in serum as target protein	Bekmurzayeva et al. (2022)
LSPR aptamer	Ochratoxin A	ochratoxin B, zearalenone	Other common mycotoxins	Lee et al. (2018)
Microfiber interferometry MIP	parathion methyl	parathion, paraoxon, fenitrothion	Have quite similar molecular structure/functional groups	Shrivastav et al. (2019)
Excessively tilted fiber grating In-house Newcastle disease virus (NDV) monoclonal antibodies	NDV	avian influenza virus and NDV-blank allantoic fluids	Other type of virus and Other virus’s allantoic fluid (prepared as the NDV allantoic fluid)	Luo et al. (2018)
Strategy 2: using a no-ligand sensor				
Ball resonator CD44 antibodies	CD44 protein	No antibody surface	To see if the signal change is due to protein binding onto antibody immobilized on the sensor	Bekmurzayeva et al. (2022)
Strategy 3: first incubate with increasing concentrations of target followed by high concentration of control(s)				
SMFC goat anti-SARS-CoV-2 IgG antibody	SARS-CoV-2 IgG	human IgG, SARS-CoV-2 IgM	Injecting higher (20 x higher than highest target analyte) concentration of control analytes	Xu et al. (2021)
SPR-POF Estrogen Receptor alpha protein	Estradiol	perfluorooctanoic acid solution	136 x higher concentration of control *Only the binding events disrupt the light modes in the POF, causing resonance wavelength shifts*	Cennamo et al. (2024)

pDNA, single-stranded probe DNA; scDNA, single-stranded complementary DNA; SMFC, single-multi-mode fiber optic coupler.

A highly specific detection of scDNA using an OFB in a microfluidic chip was demonstrated (Hu et al., 2023) where dual parameter detection of DNA hybridization and temperature using an optofluidic FBG and micro-structured optical fibers (MOF) with a single hole and dual core was demonstrated.

While the glucose/galactose binding protein (GGBP) protein is highly specific for glucose, high concentrations of fluorescent compounds near the read area can interfere with optical signal transduction, unlike electrochemical sensors. *In vitro* data demonstrated that this interference was due to direct exposure to common serum interferents. However, *in vivo* sensing, where the protein is contained within a biocompatible matrix with size-exclusion properties and concentrated near the optical fiber, is likely to reduce such interference. Additionally, increasing the GGBP protein level can enhance acrylodan emission, potentially eliminating tetracycline interference at most physiologically relevant concentrations (Weidemaier et al., 2011).

Cennamo et al. proposed an SPR biosensor which exploits specific sensing due to the device itself along with specificity of the MIP. The proposed device leverages the variation of light modes in a multimodal POF, part of the SPR platform, caused by target-receptor interactions in microholes. This differs from traditional SPR-POF-ER sensors, which rely on the RI variation of the dielectric. The receptor-analyte binding in the microholes alters the optical properties of the POF, affecting the SPR phenomena. This method, utilizing bioreceptors in an aqueous solution, is expected to achieve a lower LOD compared to MIP-based devices due to higher affinity of bioreceptors in a homogeneous phase (Cennamo et al., 2024).

## 5 Towards clinical application of packaged OFB

### 5.1 Testing in complex biological fluids

Most biosensors are tested in simpler environments like PBS (Ribaut et al., 2017; Zhou et al., 2018; Kaushik et al., 2019; Bekmurzayeva et al., 2021; Niu et al., 2022; Hu et al., 2023). But for a more accurate reflection of their performance in real-world conditions, it is crucial to evaluate them in complex biological fluids such as serum and urine. This approach helps in understanding how these sensors would behave in a natural biological context, where a multitude of factors can affect their functionality. Studies were conducted using diluted or undiluted serum/plasma to simulate a more realistic biological environment, thereby enhancing the relevance and applicability of the findings to actual physiological conditions (Luo et al., 2018; Bekmurzayeva et al., 2022).

The biosensor showed LOD of 0.35 μg/mL in undiluted plasma and achieved a total time-to-result of 12 min (Qu et al., 2022). Another biosensor detected CRP with an LOD of 0.009 mg/L in serum in a label-free manner (Aray et al., 2016). Some studies used artificial biological fluids for testing the performance of biosensors (Qiao et al., 2022; Zhou et al., 2022; Han et al., 2023; Kundu et al., 2024) which offer such benefits in biosensing studies as the capacity to regulate and establish uniform test conditions, hence guaranteeing consistency across the investigations. The sensor probe was tested for p-cresol concentrations ranging from 0 μM to 1,000 µM in artificially prepared urine to mimic real application scenarios. Artificial urine was prepared with specific components and adjusted to a pH between five and 7, with p-cresol samples homogenized within it. The results showed that the peak absorbance wavelength shift varied with the pH of the analyte, with maximum interaction efficiency at pH 6 (Usha and Gupta, 2018).

### 5.2 Testing *in vivo* and using *in vivo* mimicking setups

Dynamic and continuous measurements are crucial for packaged biosensors to accurately capture real-time changes in biological samples. This factor is especially important in situations driven by fast biological processes or where sample characteristics might vary rapidly. To enable such measurements, several pumping methods are utilized to guarantee a consistent and regulated movement of liquids through the biosensor setup. Peristaltic pumps are frequently utilized because of their capacity to deliver uniform flow rates, which is essential for preserving the accuracy of the measuring procedure (Hsu et al., 2011; Hu et al., 2023). In addition, syringe pumps have been used for their accuracy in regulating fluid flow, particularly in small quantities. Another alternative is an electronically controlled pump, which is crucial in minimizing potential environmental factors during the measurement of bio-samples (Guo et al., 2014).

Measuring biomarker levels in flow conditions presents a possibility for continuous monitoring of biomarkers in clinical environments and/or has potential for detecting circulating biomarkers which are found in low concentrations. The presence of CD44 in diluted serum was measured in a blood flow mimicking experiments using a ball resonator sensor inserted into a catheter (Bekmurzayeva et al., 2022). The packaging was later improved in a follow-up work by the authors (Myrkhiyeva et al., 2024). To replicate the dynamic flow of blood through a vein, *in vitro* measurements of CD44 protein were performed using a syringe pump at a flow rate of 20 mL/min via a tube with a diameter of 1 mm. The small size of optical fibers enabled their integration into small packaging, which is essential for applications such as embedding them in catheters for analyzing dynamic blood flow.

For monitoring of chemotherapy-induced apoptosis, *in vitro* studies involved a biomimetic 3D cell distribution simulator, while *in vivo* experiments utilized MDA-MB 231 xenografts in mice (Fan et al., 2016). This configuration enabled the evaluation of the biosensor’s efficacy in both controlled research environments and live animal models for the detection of cell death induced by chemotherapy. The detection process consisted of insertion of the sensor probe into the tumor and sequential administration of FluoMb and FM 1–43. The apoptotic activity is calculated based on the fluorescence ratio. The sensor successfully distinguished between control and chemotherapy-treated groups.

Plasmonic immunosensor showed specificity and selectivity to cytokeratin 17 inside a porous polyacrylamide gel matrix, and its effectiveness was confirmed by analyses of human lung biopsy (Ribaut et al., 2017). This indicates a notable advancement in the field of minimally invasive *in vivo* medical diagnostics and the identification of tissue biomarkers. This showed novel opportunities for the *in situ* and on-line identification of biomarkers in tissues, which is essential for non-invasive medical diagnosis and research.

Packaged OFB was also explored in theranostics when an innovative method for detecting cancer *in vivo* and treating employing fiber-optic interstitial needles was proposed (Ran et al., 2022). The fiber-optic needles are comprised of optical fibers with small diameters measuring in the range of several hundred microns. The fibers are arranged in close contact inside a syringe needle used for commercial purposes, with a diameter that is strictly controlled to be less than 1 mm. This design allows for easier interstitial navigation between tissues. The sensor was engineered for the purpose of detecting cancer *in vivo* and facilitating photothermal treatment. The method exhibited successful tumor therapy, resulting in substantial suppression of tumor development and necrosis in the treated mice as compared to the control groups. The temperature was measured with a FBG that was engraved in the rare-earth-doped fiber. This FBG functions as a comprehensive and rapidly responsive temperature monitor, offering immediate feedback during photothermal therapy.

*In vivo* continuous glucose monitoring was conducted in live rats using an optical fiber based LSPR sensor integrated with a µD probe to prevent fouling. The µD probe filtered larger molecules, allowing only glucose to pass through for detection. The sensor was calibrated against manually collected blood glucose measurements, demonstrating linear response and stability, with an overall sensitivity of 0.0354 a. u./mg/dL and an LOD of 50.89 mg/dL (Kundu et al., 2024).

### 5.3 Testing using clinical samples

Only a subset of discussed biosensors were tested using real clinical samples. The clinical application of the FRMB was demonstrated by measuring SARS-CoV-2 IgM and IgG antibodies in spiked serum samples, achieving recoveries of 80.6%–119.7% and 80.2%–120.1% with relative standard deviations below 9.6%. This method proved capable of accurately detecting these antibodies in serum samples with minimal interference, offering a simpler, faster, and more cost-effective alternative to traditional methods like ELISA and lateral flow assays. The proposed biosensor demonstrated excellent clinical performance, showing only slight redshifts for *Toxoplasma gondii* negative serum samples and significant redshifts for protozoa positive serum samples, indicating its high specificity. This specificity is due to the binding of *T. gondii* antibodies in the positive samples with antigens on the biosensor surface. The sensor’s ability to be reused after HNO_3_ treatment highlights its excellent repeatability and recovery, making it highly promising for biosensing applications (Chen et al., 2023).

### 5.4 Multiple analyte/parameter sensing

Multiplexing offers the advantage of obtaining data for various analytes simultaneously and minimizes errors by reducing the need for extensive sample handling (Kasera et al., 2014) In order to examine the ability of the microfluidic-integrated D-shaped optical fiber SERS probe to detect several molecules, a mixture of three solutions (malachite green oxalate, crystal violet, and Rhodamine 6G) were introduced into a multiplexed microchannel via various inlets, and Raman signals were acquired in the detecting region resulting in clear peaks for each of the three molecules (Bo et al., 2023).

Sensing multiple parameters or having multiple sensing points on one sensor is also a useful characteristic for a biosensor. A study introduced a novel catheter structure utilizing an FBG optical fiber sensors for urodynamic analysis, offering potential for advanced diagnostics and multiple sensing points within the bladder while ensuring biocompatibility and high accuracy (Poeggel et al., 2015). A soft biosensor that can concurrently measure the respiratory (RR) and heart (HR) rates was also presented (Lo Presti et al., 2022). A fiber optic skin-interfaced biosensor, or “smart patch,” can measure local ribcage strain brought on by respiration and heartbeat to estimate HR and RR. This was reported to be the first smart patch for cardiorespiratory monitoring that is skin-mountable and based on FBG. Another on-chip optofluidic sensing platform with packaged OFS showed high sensitivities to temperature, concentration, and RI (Li et al., 2022). Although many studies claim the multiplexing capability of sensors and/or packaging, only a limited number of studies were able to demonstrate this capability.

### 5.5 Real-time monitoring/detection

A highly sensitive sensor with real-time detection capabilities is essential for microfluidic chip applications in biochemical experiments, such as monitoring glucose concentration in a microfluidic chip with multiple inlets and reaction tanks (Tyagi et al., 2018; Li et al., 2022). Flow of the sample (artificial urine containing p-cresol) through the cell allowed a constant interaction between the sensor and the target molecules enabling continuous monitoring and real-time detection (Usha and Gupta, 2018). A study with a needle-type biosensor was designed for immunological applications, with a specific emphasis on its capabilities for real-time monitoring, *in situ* measurement, and high sensitivity (Tseng et al., 2007).

To showcase its clinical application potential, real-time Fresnel reflection light intensity was monitored by FEMB for 300 s (SARS-CoV-2 IgG) or 400 s (SARS-CoV-2 IgM). The bio-probe surface was then regenerated and washed, making it ready for subsequent tests. This real-time monitoring method demonstrated high sensitivity, specificity, and stability, making it a valuable tool for COVID-19 diagnosis and population immunization evaluation (Xu et al., 2021).

## 6 Conclusion and future perspectives

To conclude, almost all types of optical fiber sensors which are capable of biosensing were embedded into packaging which range from flow cells with a simpler construction, to more sophisticated microfluidic chips, or medical devices such as catheters and needles, or wearable devices elastic bands, patches, and textiles. These platforms can serve either for preventing the breaking of the sensor and or for *in situ* application, dynamic/continuous measurement of analytes. Analysis of research on different types of packaging for optical fiber bio-sensors showcasing examples for each type of packaging is shown in Table 4. The sensors could become more portable and practical for use in clinical settings, and it would enable the development of less invasive diagnostic procedures that could be carried out at the POC. Although potential applications of the packaged optical fiber-based biosensors include making portable and/or multiplexed devices, these aspects still require much work for real clinical applications. The measurements can be improved by the ability to use smaller volumes of reagents, measure analytes in a dynamic setup, automation of some or of the enitre process of measurement.

**TABLE 4:** Analysis of research on different types of packaging for optical fiber bio-sensors.

Flow cell						
Fabrication and design of a package	Sensor/Surface	Detection	Specificity/enhancement	Reproducibility/repeatability	Sensitivity/LOD	Ref
Flow cell is made of glass. Has specific inlet and outlet channels for fluid flow	Multimode step-index SPR optical fiber immunosensor MoS_2_ nanosheets	Water and orange juice spiked with *E. coli* bacteria was detected in PBS within 15 min	Profound specificity for *E. coli*., testing with *Salmonella Typhimurium* and *Staphylococcus aureus*	high, with a strong linear relationship	Sens: 2.9 nm/1000 CFU mL⁻^1^ (3,135 nm/RIU) LOD: 94 CFU/mL	Kaushik et al. (2019)
Molding and thermal bonding techniques A single-channel, aluminum bottom and a PDMS top, Peltier element for temperature stabilization	SPR-based Side-polished multimode POF Gold	In human serum CRP was detected within 15 min	Distinct SPR responses to CRP-spiked serum compared to CRP-free serum	High, same shifts in SPR wavelength for different CRP concentrations	LOD: 0.009 mg/L	Aray et al. (2016)
Laser writing technology on PMMA plates A single-channel flow cell with a PDMS chamber for sample delivery	Optical microfiber coupler sensor Polyelectrolyte layers	In PBS cTnI was detected within 10 min	Low responses for non-specific proteins (CRP, IgG, and PSA)	Good, maximum RSD of 8.39%	LOD: 2 fg/mL	Zhou et al. (2018)
Microfluidic chip						
SU-8 photolithography Typical double-Y channel structure (130 μm × 130 μm)	WGM fiber probe PDA	In PBS Cardiac biomarker cTnI-C was detected within 30 min response time	Significant FSRRF changes for cTnI-C compared to other non-specific proteins such as PSA, CRP, IgG, and BSA.	Good, consistent responses across multiple tests	Sens: 0.991 nm/(ng/mL) LOD: 0.59 ng/mL	Niu et al. (2022)
Femtosecond laser direct writing micro/nano processing technology. PDMS and glass were used Soft zigzag microchannel connected to the single channel	Interferometer- based MOF combined with a FBG PEI	In PBS scDNA and temperature were detected within 10 s	Low responses for non-specific and mismatched sequences compared to scDNA.	Multiple independent measurements of scDNA and non-scDNA	LOD: 1.87 nM	Hu et al. (2023)
Dimensions: width 200 μm by height 150 μm UV-sensitive adhesive on both sides of the sensing element, thus delimiting a length of 20 mm	TFBG ultra-thin nanometric silver coating	Urine proteins 1.0 s per full scan by the OSA	Urine samples from healthy rats, rats with adriamycin-induced nephropathy, and treated rats were used	Multiple measurements of protein concentrations in urine samples from healthy, sick, and treated rats	Sens: 5.5 dB/(mg/mL) LOD: 1.5 × 10^-3 mg/mL	Guo et al. (2016)
Catheter						
Silicone made. Sensor is packaged to combined catheter extension set with syringe (two heat-shrink tubes fixing a rectangular two-layer strip on the sidewall of the optical fiber)	Fiber optic photoacoustic sensor	In human plasma and blood heparin short turnaround time (3 min)	Specific to heparin due to the electrostatic interaction	Good, relative standard deviations of <16.6% in whole blood in the simulated *in vivo* model	LOD: 0.17 U/mL in PBS	Zhou et al. (2022)
Ready-to-use medical device	Ball resonator (spherical tip) Silanization	CD44 in diluted serum was detected in 10 min	PSA	High: sensors exhibited a similar trend, given that they also have different sensitivity	Not so high sensitivity LOD: 4.68 a.m.	Bekmurzayeva et al. (2022)
Needle						
The design involves two optical fibers and two microcapillaries aligned within a gauge 20 hypodermic needle. The microcapillaries are used to deliver fluorescent dyes to the tumor site	Fiber optic sensor probe	In tumor in both *in vitro* and *in vivo* phospholipid conjugated Marina Blue (FluoMb) was detected within 10–15 min	Detected apoptotic activity induced by chemotherapy, distinguished by the fluorescent emission ratio of FluoFM 1–43 to FluoMb	Reproducibility: a bias lower than 16%	Sens: 1 × 10^7 cells/mL	Fan et al. (2016)
Each polymer solution was loaded into a syringe with an attached stainless steel 27-gauge blunt-tip needle (Fisnar)	WGM optical biosensors	Analyte: Submerged in buffer solutions streptavidin	The M13 bacteriophage bioreceptors provided specificity for streptavidin binding	Fibers retained functionality after the fabrication process	Sens: 0.008 nm/nM LOD: 3 nM	Hsieh et al. (2022)
Commercial syringe needle with a total diameter of less than 1 mm to aid in interstitial navigation	Hypoxia-sensitive fluorescent fibers and rare-earth-doped fibers	NTR, a hypoxia biomarker, was detected within 20 s (full detection time 15 min)	Specificity achieved through hypoxia-sensitive fluorescent probes	High: consistent results in multiple *in vivo* and *in vitro* experiments	LOD: 5 ng/mL	Ran et al. (2022)
Elastic band structure						
The elastic belt structure: sensor, piece of plastic belt, and an elastic fabric Elastic band is 80–90 cm long and 10 cm wide	D-shaped plastic optical fiber	Respiratory rate in abdomen	Wavelet noise reduction technology	Respiratory rate of 6 participants	Highly sensitive	Wang et al. (2021b)
Fabric - wearable patterned Janus textiles						
Hydrophilic properties on its two sides. The side in contact with the skin is superhydrophobic. PDMS layer: reservoir for storing the extracted sweat	Microstructured grapefruit optical fiber	Urea and lactic acid in sweat. Reaction time was 3 min	SERS: decoupled sweat collection and detection	The uniformity and repeatability of the SERS substrate were confirmed with an RSD lower than 7%	Highly sensitive LOD: 0.1 mM	Han et al. (2023)
Smart Patch						
The overall dimensions of the smart patch are 40 mm × 25 mm × 2 mm Flexible matrix (i.e., silicone layer) layered between two fabric liners to obtain the proposed smart patch	FBG	heart and respiratory rate	n/a	Repeatability: Strain response mechanical test was performed ten times. The results fall on a linear trend	n/a	Lo Presti et al. (2022)

LOD, limit of detection; PDMS, polydimethylsiloxane; PMMA, polymethyl methacrylate; SPR, surface plasmon resonance; *Escherichia coli*–*Escherichia coli*; PBS, phosphate-buffered saline; CRP–C-reactive protein; MoS₂–molybdenum disulfide; POF, plastic optical fiber; cTnI-C, cardiac troponin I complex; IgG–immunoglobulin G; PSA, prostate-specific antigen; RSD, relative standard deviation; BSA, bovine serum albumin; PDA, polydopamine; HGMS, hollow glass microsphere; WGM, whispering gallery mode; scDNA, single-stranded DNA; MOF, micro-structured optical fiber; TFBG, tilted fiber Bragg grating; PEI, polyethyleneimine; OSA, optical spectrum analyzer; NTR, nitroreductase; SERS, surface-enhanced Raman scattering.

The development of microfluidic systems integrated with OFS, offers important benefits in biological and diagnostic applications. These microfluidic tools improve sensitivity and accuracy by allowing exact control and change of fluids at the microscale. By automating and downsizing tests, microfluidic devices have the potential to transform biosensing by making them transportable and affordable. These devices can increase sensitivity and consistency by controlling sample flow and delivery to biosensors. Additionally, they improve thermal management and lessen temperature gradients while lowering the required reaction volumes. Integration of biosensors with a microfluidic chip can bring it closer to develop a POC devices. POC devices are fast, robust, non-invasive devices which are simplified and miniaturized for diagnostic purposes (Hayes et al., 2018). POC can be used in such settings as clinical laboratories, doctor’s office and ultimately at home. The low cost of the devices will make them useful for improved screening of a wider population, monitoring disease recurrence and better surveillance of cancer treatment (Soper et al., 2006).

In the nearest future, an advancement in lab-in-a-needle technology is expected which will involve the creation of smart needle-based devices that have exceptional levels of functionality, integration, and miniaturization. It is anticipated to have a transformative impact on the management of cancer, particularly in localized therapies, by allowing the monitoring of disease development and progression. Advanced fiber-based systems incorporated into hypodermic needles will enable the monitoring of cancer biomarker levels in close contact to tumors. The use of lab-in-a-needle technology is expected to stimulate progress in both academia and industry, resulting in the development of innovative local biopsy instruments and the discovery of novel biomarkers (Liao et al., 2019).
